# Experimental Evaluation of Wi-Fi and BLE Smart Particles in a Rotating Drum: Link-Budget-Normalised RSSI Characterisation and IMU Validation of the Wi-Fi Particle

**DOI:** 10.3390/s26175481

**Published:** 2026-08-29

**Authors:** Nancy Gulati, Tahir Jauhar, Gabriel Lodewijks, Michael Carr, Craig Wheeler

**Affiliations:** School of Engineering, College of Engineering, Science and Engineering, University of Newcastle, Newcastle 2308, Australia; nancygulati91@gmail.com (N.G.); gabriel.lodewijks@newcastle.edu.au (G.L.); michael.j.carr@newcastle.edu.au (M.C.); craig.wheeler@newcastle.edu.au (C.W.)

**Keywords:** smart particles, wireless sensing, RSSI, link budget, fading statistics, rotating drum, granular dynamics, industrial IoT, sensor selection

## Abstract

**Highlights:**

**What are the main findings?**

- Under rotation, the Wi-Fi particle holds an RSSI standard deviation of 1.96 dB, against 6.27 and 6.58 dB for the two BLE particles.
- Two BLE particles of the same protocol class differ by 23.9 dB, of which a maximum of 4 dB is attributable to power transmission, and particles logged simultaneously through one receiver fluctuate independently.

**What are the implications of the main findings?**

- Link quality inside a rotating drum is a property of the individual device rather than of its protocol class, so candidate particles must be characterised in situ instead of being selected from datasheets.
- The preferred particle changes once battery endurance carries more than 0.35 of the decision weight, which sets the design trade-off for instrumenting tumbling mills and granulation drums.

**Abstract:**

Wireless sensing inside rotating industrial machines is challenging due to signal attenuation, multipath propagation, and continuous sensor motion. Smart particles equipped with wireless communication and inertial sensors provide a promising approach for monitoring such systems. However, the reliability of wireless signal transmission under rotational dynamics remains insufficiently understood, and systematic approaches for sensor selection are lacking. This paper presents a link-budget-normalised experimental characterisation of three commercial smart particles, namely MetaMotionS (BLE), WitMotion BLE, and WitMotion Wi-Fi, in a bare 300 mm diameter by 310 mm deep metallic drum fitted with six triangular lifters, at rest and at 16, 18, and 20 RPM, corresponding to 20.7–25.9% of the critical speed and Froude numbers of 0.043–0.067. Because raw received power conflates transmit power with channel behaviour, the comparison is expressed as excess path loss above free space together with second-order fading statistics. Two particles of the same protocol class differ by 23.9 dB, of which at most 4 dB is attributable to the transmission of power across the documented range of both radios, establishing that device implementation rather than protocol class governs the ranking. Two particles logged simultaneously through a single receiver observe one channel realisation, and the correlation between their signal fluctuations is not significantly different from zero at any speed (r = +0.064, −0.098, −0.083), indicating device-specific rather than environmental fading. Under rotation, the Wi-Fi particle holds an RSSI standard deviation of 1.96 dB against 6.27 and 6.58 dB for the two BLE particles (Welch ANOVA, *p* < 0.001; Games–Howell post hoc, all pairwise comparisons *p* < 0.001; |Cliff’s δ| > 0.96). A bounded, dimensionless multi-criteria selection procedure over link margin, signal variability, cross-speed consistency, packet delivery, and energy per delivered packet is introduced; the ranking is invariant under weighted-sum and TOPSIS aggregation but inverts once endurance carries a weight above 0.35, which quantifies the trade-off between link quality and battery life. Coupling between the wireless and motion streams is examined by folding both onto rotation phase. A rotation-locked component in RSSI is detected in one of nine sensor–speed combinations, with a maximum modulation amplitude of 0.92 dB. The RSSI logging cadence of approximately 1 Hz resolves the drum fundamental but lies below the Nyquist requirement for the dominant motion band at 0.91–1.04 Hz, so joint wireless–motion studies of rotating machinery require RSSI logging at 5 Hz or above on a clock shared with the inertial unit.

## 1. Introduction

Rotating machines such as granulation drums, ball mills, rotary kilns, and mixing vessels are widely used in industrial processes, including mineral processing, pharmaceuticals, chemical manufacturing, energy production, and transportation [1,2]. These systems are essential for material processing and energy conversion. Reliable operation is important for maintaining productivity, efficiency, and safety. Even minor faults can cause unplanned downtime, increase maintenance costs, and create safety risks. This highlights the need for effective monitoring solutions that capture internal system behaviour [2].

Monitoring the internal behaviour of rotating machines remains challenging due to limited physical accessibility, harsh operating conditions, and continuous motion. Conventional wired sensing approaches are often impractical in such environments. In this context, smart particles, which are compact self-contained sensing devices equipped with inertial sensors and wireless communication modules, have emerged as a promising approach for probing the internal behaviour of rotating equipment without intrusive modifications. Instrumented particles have been used to measure motion and impact behaviour in tumbling mills and related granular systems, demonstrating their potential for in situ monitoring of particle dynamics inside rotating process equipment [3,4].

A key limitation in smart particle deployment is the reliability of wireless communication within rotating metallic enclosures. Signal attenuation, multipath propagation, scattering, and absorption caused by metallic components can significantly degrade signal quality. In addition, continuous particle rotation introduces orientation-dependent antenna effects and time-varying propagation paths, which lead to fluctuations in received signal strength. Similar communication challenges have been reported in studies of wireless monitoring in rotating machinery and complex electromagnetic environments [5,6]. Understanding the impact of these dynamic conditions on wireless signal behaviour is therefore essential for achieving reliable real-time monitoring using smart particles.

To evaluate wireless communication performance in such environments, the received signal strength indicator (RSSI) is widely used as a practical metric for assessing link quality [6,7]. RSSI measurements help show how strong and stable a signal is. They are also affected by the surrounding environment, the orientation of the sensor, and motion during operation. However, RSSI data are inherently noisy and can exhibit significant variability in dynamic environments such as rotating machines. Experimental studies are therefore needed to better understand how rotational motion and particle dynamics influence wireless signal behaviour.

In addition to wireless communication analysis, smart particles typically incorporate inertial measurement units (IMUs) to capture motion information such as acceleration and angular velocity. These measurements provide valuable insight into particle motion behaviour inside rotating equipment. However, IMU measurements are subject to sensor noise, bias, and drift, which can build up over time and affect motion interpretation if the sensor is not properly calibrated and validated [8]. Reliable calibration procedures are therefore necessary to ensure that IMU measurements accurately represent the physical motion of the instrumented particle.

Despite recent advances, several research gaps remain. First, there is limited experimental comparison between BLE-based and Wi-Fi-based smart particles operating inside rotating machines under controlled rotational speeds. Second, the relationship between particle motion dynamics captured by IMU measurements and wireless signal behaviour inside rotating metallic environments remains insufficiently explored. Finally, experimental studies that jointly examine particle motion dynamics and wireless signal variability in rotating environments remain relatively scarce.

This study addresses these gaps through a comprehensive experimental investigation of wireless communication performance and particle motion dynamics inside a rotating granulation drum used as a representative rotating machine. It further examines how particle motion characteristics influence wireless signal variability, enabling a deeper understanding of signal behaviour in dynamic rotating environments. A sensor selection protocol based on RSSI stability, signal robustness, and motion sensing reliability is defined and applied to systematically evaluate and compare sensor performance. RSSI measurements are collected under stationary and rotational operating conditions using BLE-based and Wi-Fi-based smart particles with data received from an external ESP32-based receiver. In addition, IMU measurements from a Wi-Fi-enabled smart particle are analysed to characterise particle motion behaviour inside the rotating drum. A pendulum experiment combined with ground-truth tracking using a video camera is used to calibrate and validate the IMU measurements. Finally, experimentally measured motion behaviour is analysed using calibrated IMU signals to better understand particle dynamics under rotation.

The main contributions of this work are summarised as follows:

- Application of a sensor selection protocol for systematic comparison of smart particle performance in rotating environments;
- Experimental comparison of BLE and Wi-Fi smart particle RSSI performance inside a rotating drum environment;
- Statistical analysis of RSSI behaviour under stationary and rotational operating conditions;
- Pendulum IMU calibration and validation using video-based ground-truth tracking;
- IMU characterization of particle motion dynamics at different drum rotational speeds;
- Link-budget normalisation of the wireless comparison, separating transmit power from channel behaviour, together with a paired same-instant comparison of two simultaneously logged particles;
- A bounded, dimensionless multi-criteria selection procedure incorporating endurance, with explicit weight sensitivity analysis identifying where the recommendation changes;
- A rotation-phase-resolved analysis of coupling between the wireless and motion streams, and a quantitative statement of the logging rate required to recover sample-level coupling.

## 2. Related Work

Physics-based analytical models were used in early monitoring and analysis of rotating machines [9]. Classical methods employ state-space representations, equivalent circuit models, and differential equations to describe the dynamic behaviour of rotating electrical machines and to support fault diagnosis and performance estimation [10,11]. These approaches laid the theoretical foundation for understanding machine dynamics and failure mechanisms. However, their practical application often requires extensive manual modelling and tuning. They also showed limited ability to adapt to non-stationary operating conditions and to provide real-time access to internal machine states [12].

To address these limitations, sensor-enabled monitoring techniques have become increasingly popular. Vibration and signal processing approaches were widely adopted to infer machine condition using externally mounted sensors [13]. While these methods work well in many situations, they still rely on indirect observations when internal dynamics are important. As a result, embedded sensing approaches, including instrumented components and smart particles, have gained attention for directly capturing internal motion and interactions within rotating systems.

One of the earliest examples of this approach is the wireless instrumented sphere, which demonstrated the feasibility of embedding inertial sensors and wireless communication modules within a rotating body to capture acceleration and motion-related information in real time [14]. Building on this concept, instrumented grinding media have been used in tumbling and rotating process equipment to infer internal conditions such as load behaviour and impact regimes from acceleration measurements [15,16]. These studies clearly show the value of smart particles for in situ monitoring. However, their primary emphasis has been on mechanical measurements, with limited analysis of wireless communication performance itself.

In parallel, wireless communication systems have emerged as an enabling technology for scalable and flexible monitoring of industrial machinery. Wireless sensors reduce installation complexity and are particularly attractive for rotating equipment where cabling is impractical. Nevertheless, wireless propagation in metallic and enclosed environments is known to be challenging due to severe attenuation, reflection, and multipath effects [17]. Experimental studies conducted in dense metal and compartmentalised environments have shown that link quality can vary significantly depending on geometry and surrounding structures, highlighting the need for environment-specific validation [18].

RSSI has been widely adopted as a practical metric for assessing wireless link quality. In wireless sensor networks and IoT systems, RSSI is commonly used to evaluate communication reliability, optimise network performance, and support distance estimation [19]. However, RSSI measurements are highly sensitive to environmental factors and are known to exhibit substantial variability, particularly in multipath and obstructed environments [20]. These limitations suggest that RSSI behaviour must be carefully characterised before it can be reliably used in more complex scenarios such as rotating machinery. Only a limited number of studies have explicitly investigated wireless communication behaviour in complex and dynamic industrial environments. Recent work has explored the use of sensor networks to support near-real-time digital twin fidelity, highlighting the importance of reliable data acquisition under varying operating conditions [21]. However, such studies do not consider smart particle sensing or the role of embedded sensor motion in shaping wireless signal variability.

Alongside wireless considerations, inertial measurement units (IMUs) are a key component of smart particle systems. Numerous studies have investigated the performance characteristics of MEMS IMUs, thereby identifying issues such as sensor noise, bias instability, and drift [22,23]. These irregularities can affect the accuracy and interpretability of motion measurements, especially in dynamic and rotating applications. Despite this, the relationship between IMU–derived motion dynamics and wireless signal variability has received relatively little attention in the literature. Previous studies have extensively investigated particle motion and granular flow behaviour in rotating drums using both experimental and modelling approaches [24,25,26,27,28,29,30]. These works provide valuable insight into particle trajectories and flow regimes. However, they do not consider wireless communication behaviour or the interaction between particle motion and signal variability.

Despite these advances, most existing studies examine wireless communication performance and motion sensing separately. Systematic experimental comparisons of different wireless technologies, such as BLE and Wi-Fi under controlled rotational conditions, remain limited. In addition, only a small number of studies investigate how the motion of a sensor embedded inside a rotating machine affects wireless signal behaviour. These limitations highlight the need for experimental investigations that evaluate wireless communication performance together with particle motion using smart sensors. The present study addresses the stated needs by analysing the measured motion of the embedded sensor using calibrated IMU data and experimental validation. A parallel body of work on integrated sensing and communication co-designs waveforms, beamforming, and network resources so that a single transmission serves both functions, including recent treatments of reconfigurable-surface-assisted full-duplex networks under secrecy and sensing constraints [31]. That literature optimises the network given a tractable propagation model. The problem addressed here is complementary and prior to it: a smart particle carries sensing and communication as two independent functions on one commercial node, and what is unknown is whether the radio link itself survives being sealed inside a rotating metallic enclosure.

A summary of related studies and their main limitations compared with the proposed approach is presented in Table 1. The comparison highlights that existing studies typically focus either on wireless communication or motion sensing, whereas this work integrates both aspects and explicitly examines their interaction.

## 3. Materials and Methods

### 3.1. Experimental Testbed and System Architecture

The experimental setup was developed to investigate wireless communication performance and motion sensing behaviour of smart particles operating inside a rotating machine environment. A rotating drum testbed was used to reproduce representative industrial conditions, including continuous rotation and the presence of metallic structures that influence wireless signal propagation. Smart particle sensors were placed inside the rotating drum while a wireless receiver located outside the system collected the transmitted data.

Each smart particle is equipped with an IMU and a wireless communication module. During operation, the IMU records motion-related measurements as the particle moves within the rotating drum while the sensor simultaneously transmits wireless packets to the external receiver. The received signal strength indicator associated with each packet is recorded and used as the primary metric for evaluating wireless communication performance. One sensor is used per test at a time.

**Figure 1:**
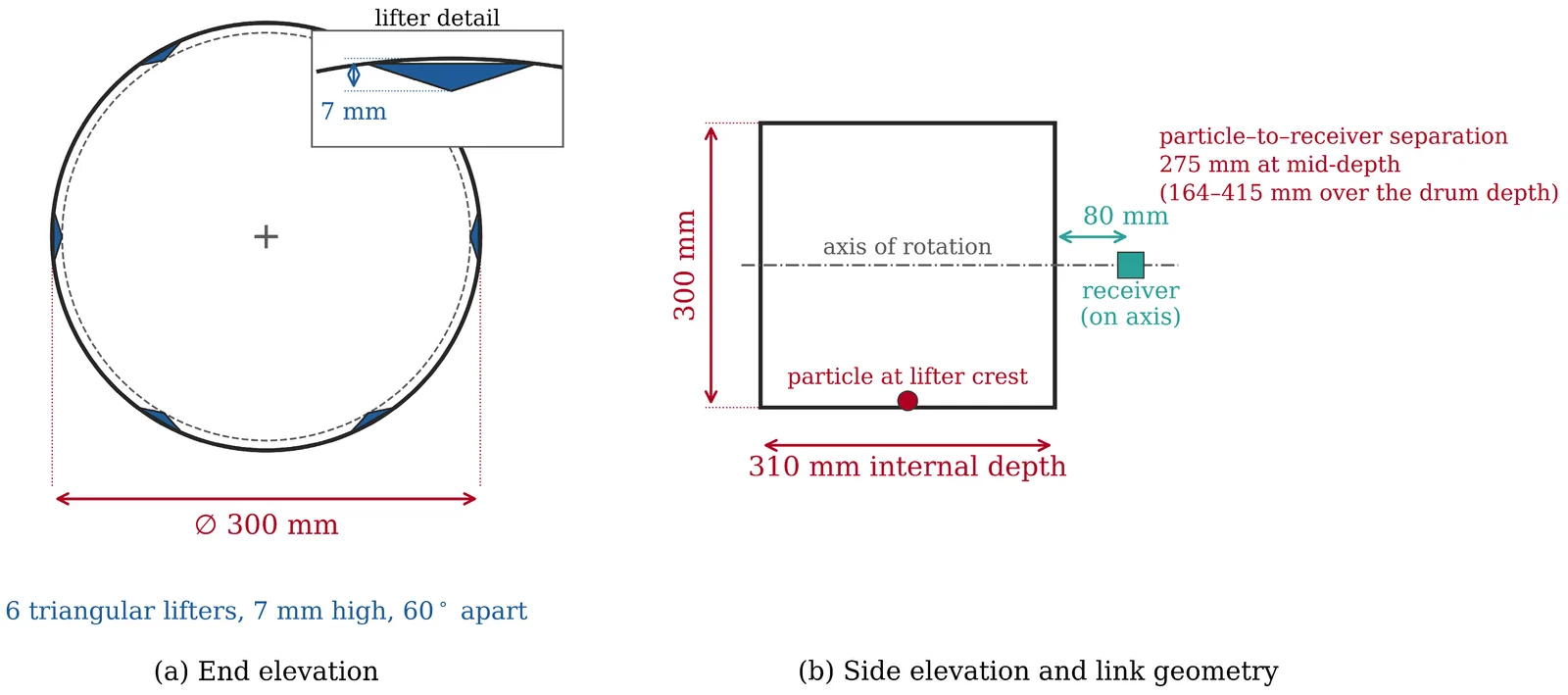
Rotating drum testbed. (**a**) End elevation showing the 300 mm bore, the 6 triangular lifters, and the lifter crest circle, with a magnified detail of the lifter section. (**b**) Side elevation showing the 310 mm internal depth, the receiver on the axis of rotation at its measured axial offset, and the resulting particle-to-receiver path length.

This configuration enables simultaneous analysis of particle motion and corresponding wireless signal behaviour under both stationary and rotating operating conditions. Figure 1 illustrates the overall system architecture delineating the embedded smart particle within the rotating drum and the external wireless data acquisition system.

The drum has an internal diameter of 300 mm and an internal depth of 310 mm, giving an internal volume of 21.9 L and a depth-to-diameter ratio of 1.03. The shell carries 6 equally spaced lifters of triangular section standing 7 mm proud of the bore, at 60° intervals, which prevent a particle from sliding continuously against the wall and impose a lifting and release cycle once per lifter passage. The geometry is given in Figure 1 and summarised in Table 2.

The receiver is mounted on the axis of rotation, 80 mm beyond the end plate. Its position was determined by the space available in the workshop rather than selected to optimise link quality. Placing the receiver on the axis has a consequence used later in the analysis: the geometric path length from the receiver to a particle at a fixed radius does not change as the drum turns, because rotation carries the particle around the receiver rather than towards or away from it. Path length varies only with axial position, from 164 mm at the near end plate to 415 mm at the far end, with a mid-depth value of 275 mm. Any rotation-locked variation in received power therefore cannot be a range effect.

### 3.2. Smart Particle Sensors

**Figure 2:**
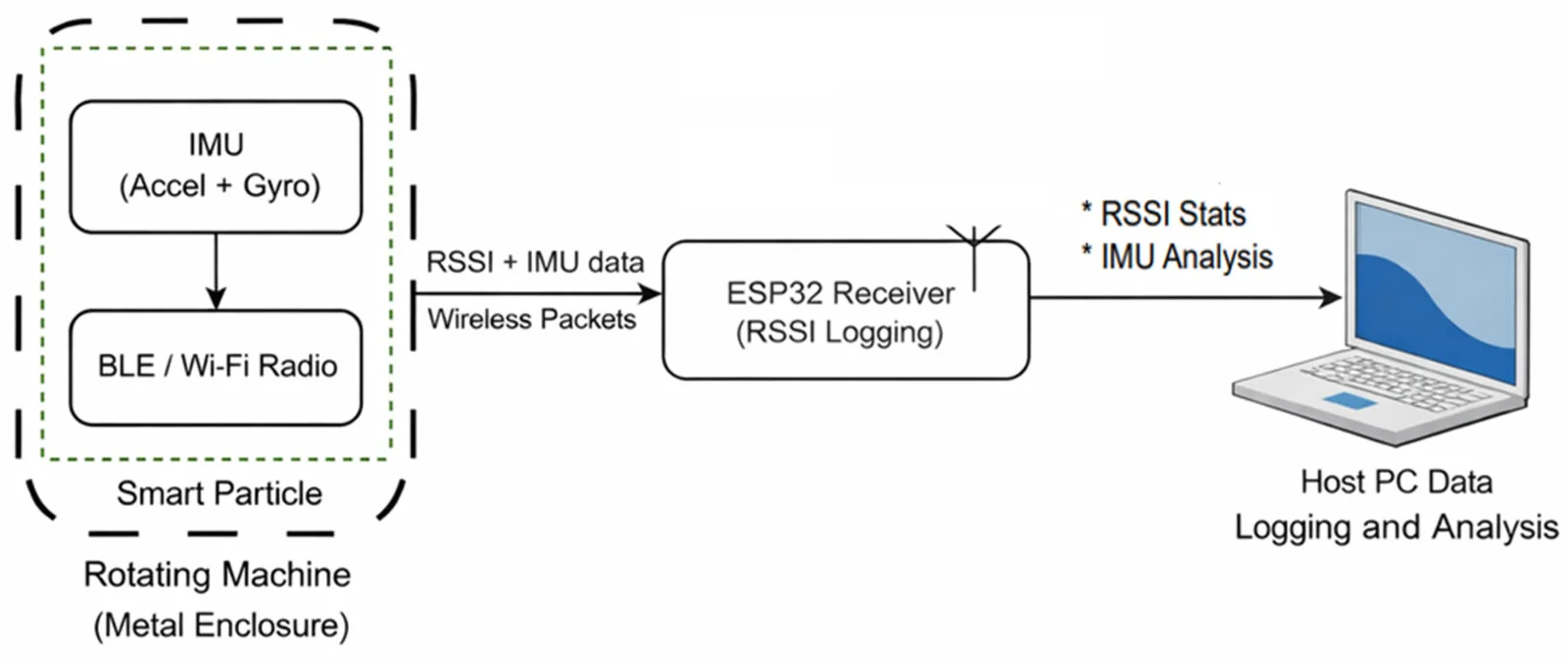
System-level architecture of the smart particle monitoring framework.

Three commercially available smart particle sensors were evaluated in this study, namely MetaMotion S (MbientLab Inc., San Francisco, CA, USA), WitMotion BLE (WitMotion Shenzhen Co., Ltd., Shenzhen, China), and WitMotion Wi-Fi (WitMotion Shenzhen Co., Ltd., Shenzhen, China). These sensors were selected to enable a comparative assessment of Bluetooth low energy (BLE) and Wi-Fi communication technologies under identical operating conditions in a rotating environment. Two sensors operate using BLE communication, while one sensor uses Wi-Fi communication. All sensors are equipped with onboard IMUs capable of measuring acceleration and angular velocity, enabling simultaneous analysis of particle motion and wireless communication performance. The system level architecture remains consistent for all the sensors as shown in Figure 2. The Metamotion S sensor includes a 9-axis IMU and supports BLE communication [32]. The WitMotion BLE and WitMotion Wi-Fi sensors also incorporate inertial sensing units with BLE and Wi-Fi communication capabilities, respectively [33,34].

The technical specifications of the evaluated sensors, obtained from the respective manufacturer datasheets, are summarised in Table 3. These specifications include sensing ranges, resolution, data rates, and communication characteristics that influence sensor performance under dynamic operating conditions.

Transmit power, receiver sensitivity, radio system-on-chip, antenna type, and battery capacity are taken from the manufacturers’ published documentation.

The sensors differ in their communication protocols, sensing capabilities, and data acquisition characteristics. Variations in transmission mechanisms, sampling rates, and sensor resolution may influence RSSI behaviour and motion sensing performance within the rotating drum environment.

**Figure 3:**
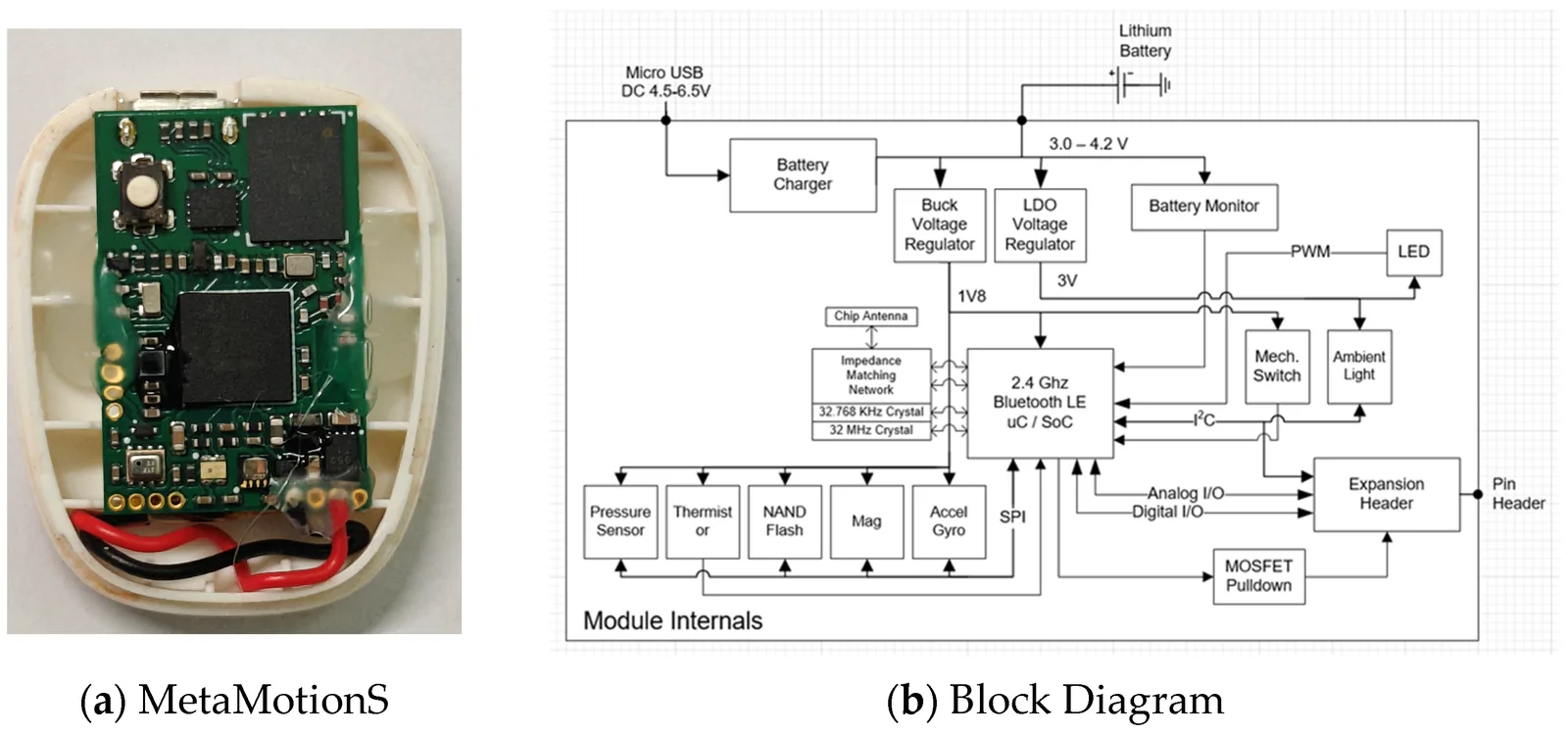
Internal layout of a sensor in a smart particle.

The MetaMotionS particle was logged in a separate session from the two WitMotion particles. The WitMotion BLE and WitMotion Wi-Fi particles were served by a single receiver instance during the rotating runs, so their records share a common receiver clock and sample the same channel realisation. That pairing is exploited in Section 4.2.1. Figure 3a illustrates the internal layout of MetaMotionS, with Figure 3b showing the block diagram mentioning the integrated IMU, wireless communication module, and power source.

### 3.3. Wireless Data Acquisition and Receiver Configuration

Wireless signal measurements were collected using an external receiver positioned outside the rotating drum. The receiver was implemented using an ESP32 microcontroller configured to receive wireless packets transmitted by the smart particle sensors. During each experiment, the receiver continuously monitored incoming data packets and recorded the associated received signal strength indicator values.

The receiver was connected to a monitoring computer where the received data were logged for further analysis. Each recorded packet included the sensor identifier timestamp and RSSI value, which enabled detailed evaluation of wireless signal behaviour during both stationary and rotating operating conditions.

To ensure reliable measurements, the receiver was placed at a fixed location relative to the rotating drum throughout all experiments. This configuration allowed consistent observation of wireless signal variations caused by drum rotation sensor motion and the surrounding metallic environment. Figure 3 shows the schematic diagram depicting the position of the wireless receiver and its distance from the rotating drum.

Received power was logged at a median inter-packet interval of 1.02 s (0.98 Hz), timestamped on the receiver’s monotonic millisecond clock. The receiver reports RSSI with a quantisation step of 1 dB. No reference transmitter calibration was carried out, so the absolute received power carries a fixed offset that is not determined here; published characterisations of this receiver family report deviations in the order of 5 dB against calibrated instruments. Because the offset is a property of the receiver and is common to every measurement it makes, it cancels out in the between-device comparisons and in the variance-based metrics on which the conclusions of this work rest and affects only statements about absolute level. The ambient noise floor at the receiver position was not measured; the receiver’s own sensitivity, −94 dBm, is therefore used as a lower bound in its place, and every link margin reported below is correspondingly an upper bound.

Under stationary conditions, the Wi-Fi particle was logged at a 2.99 s cadence rather than 1.02 s, so its stationary variability is not directly comparable with the rotating cases and is identified as such where it appears. Three of the four Wi-Fi records end in a contiguous block of rows carrying no received power value, while the simultaneously logged BLE stream continues normally. Such a block is excluded from the packet statistics only when it satisfies three conditions: it is contiguous, it terminates the record, and every preceding row in the same record carries a valid value. A block meeting all three is treated as a termination of logging rather than as a loss on the link on two grounds: a loss on the link would not leave the concurrently logged BLE stream unaffected, and each truncated record stops at exactly 500 valid samples, which is a property of the logging application rather than of the channel. On this criterion, 93, 11, and 20 trailing rows are excluded at 16, 18, and 20 RPM, respectively, leaving 514, 500, and 500 delivered packets; the stationary Wi-Fi record contains no such block. The excluded counts are reported in the final column of Table 4, so the loss rates can be recomputed on the opposite convention. No interior gap in any record satisfies the criterion, so no loss occurring during a record is removed by it.

Losses are inferred from gaps in the fixed logging cadence and from rows logged without a received power value; contiguous blocks that terminate a record and are preceded only by valid samples are treated as terminations of logging, excluded from the loss rate, and reported separately in the final column (Section 3.3).

### 3.4. Experimental Procedure

Experiments were conducted under both stationary and rotating conditions to isolate the effect of drum rotation on wireless communication performance and particle motion. Under stationary conditions, the drum remained static to establish a baseline for RSSI measurements. For rotating conditions, the drum was operated at predefined speeds of 16 RPM, 18 RPM, and 20 RPM.

During each experiment, a single smart particle sensor was placed inside the rotating drum while the wireless receiver remained positioned outside the system at a fixed location. Prior to data collection, the wireless connection between the sensor and the receiver was verified to ensure stable packet reception. RSSI values associated with each received packet were continuously recorded and stored on the monitoring computer for further analysis.

**Figure 4:**
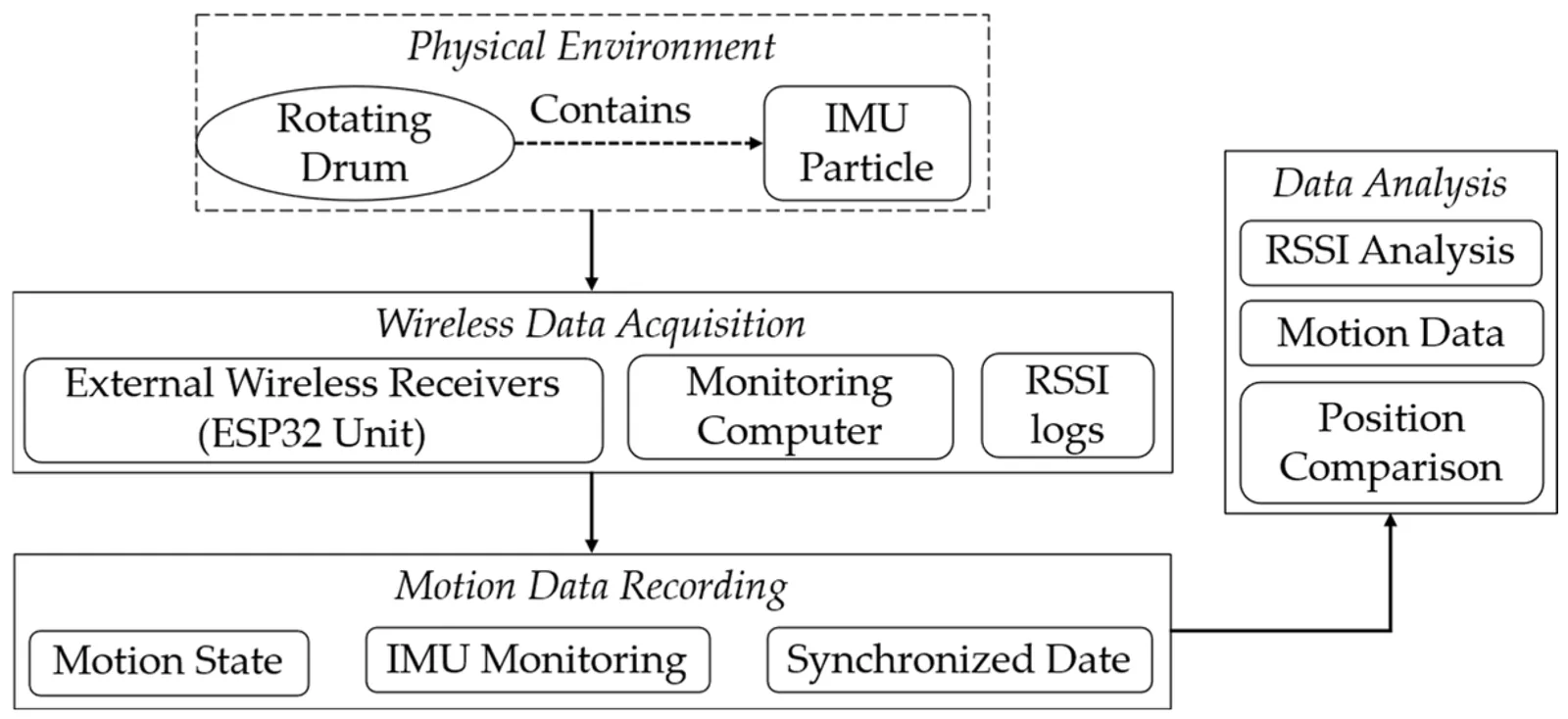
Workflow of wireless data acquisition and motion analysis.

For each operating condition, more than 500 RSSI samples were collected to ensure reliable statistical evaluation of wireless signal behaviour. The experiments were repeated for each smart particle sensor under identical conditions to enable a fair comparison. Wireless and inertial measurements were acquired in separate sessions on independent clocks. The coupling analysis of Section 4.5 is accordingly conducted at the ensemble level, using the drum revolution as the common reference. The overall workflow from sensor deployment to data acquisition and analysis is illustrated in Figure 4.

The drum was operated bare, with a single-vendor IMU sensor and no granular charge. At the speeds tested, the particle occupies 20.7 to 25.9% of the critical speed, corresponding to Froude numbers of 0.0429 to 0.0671 at the shell and 0.0409 to 0.0639 at the lifter crest. Although the speed range spans only ±12.5% about its midpoint, the Froude number varies by 56% across it, since Fr scales with the square of angular velocity. The dynamically relevant parameter therefore spans a considerably wider range than the nominal speed range suggests.

An unconstrained single particle in a bare drum does not follow a repeatable trajectory. Two independent runs were recorded at each speed and are reported separately throughout, so that run-to-run variability remains visible.

### 3.5. Sensor Selection Framework

A candidate smart particle is evaluated on five criteria, each of which is physically interpretable and separately measurable.

Link margin is the mean received power relative to the receiver sensitivity,

(1) $$M=\mu_{RSSI}-P_{sens}$$

where *μ*_RSSI_ is the mean received power in dBm and *P*_sens_ the receiver sensitivity in dBm. Referencing the level to the receiver rather than to the 1 mW datum of the dBm scale makes the criterion invariant to that choice of reference. Signal variability is the within-condition standard deviation $\sigma_{RSSI}$. Above the receiver noise floor, this quantity is largely independent of transmit power, so it characterises the channel and antenna rather than the link budget. Cross-speed consistency is the standard deviation of the per-speed mean received power, $\sigma_{speed}$. Low values indicate performance that does not depend strongly on the operating point. Packet delivery is the delivered-to-expected packet ratio, reported as a loss rate with a Wilson score interval.

Energy per delivered packet is the stored energy of the particle divided by the number of packets it delivers on one charge,

(2) $$E_{pkt}=\frac{QV}{f_{del}\;t_{run}}\;;where\;t_{run}=\frac{Q}{I}$$

with *Q* representing the battery capacity, *V* representing the nominal cell voltage, *I* representing the mean operating current, and *f*_del_ representing the measured delivered packet rate. Charging a device for the energy it spends on packets that do not arrive makes the criterion a measure of useful work rather than of endurance alone.

Each criterion is min–max-normalised across the candidate set to a dimensionless value in [0, 1], in which unity is best,

(3) $$n_{j}=\frac{x_{j}-x_{min}}{x_{max}-x_{min}}\;\;\;\left(\mathrm{benefit}\right),\;\;\;\;\;\;n_{j}=\frac{x_{max}-x_{j}}{x_{max}-x_{min}}\;\;\;\left(\cos t\right)$$

and the normalised criteria are aggregated both by weighted sum and by the TOPSIS relative-closeness index,

(4) $$S=\sum_{j}w_{j}n_{j},\;\;\;\;\;\;\;\;C^{*}=\frac{d^{-}}{d^{+}+d^{-}}$$

where *d*^+^ and d^−^ are the Euclidean distances of a candidate from the ideal and anti-ideal points in the weighted normalised criterion space. The two rules differ in their treatment of compensation: the weighted sum allows a strong criterion to offset a weak one, whereas TOPSIS penalises distance from the ideal on every criterion. Agreement between them indicates that a ranking is a property of the measurements rather than of the aggregation rule, and both are therefore reported.

Taking the Wi-Fi particle under rotation, the mean received power is −47.04 dBm against a receiver sensitivity of −97 dBm, so from Equation (1),

M = −47.04 − (−97) = 49.96 dB

and for MetaMotionS, M = 11.83 dB. A larger margin is better, so with three candidates spanning 11.8 to 50.0 dB, the normalised link margin of the Wi-Fi particle is unity, and that of MetaMotionS is zero. The sign convention is worth making explicit because received power is negative on the dBm scale: a stronger signal is a less negative number, hence a larger margin above sensitivity and a larger normalised score. A criterion defined directly on |*μ*_RSSI_| would invert this ordering, which is why the margin form of Equation (1) is used.

A candidate must first satisfy a positive link margin with an implementation reserve appropriate to the installation. Above that threshold, reliability is governed by fade depth and outage rather than by mean level, so variability should carry the larger weight. The relative weighting of link quality and endurance is examined in Section 4.2.3, where the weighting at which the preferred candidate changes is identified.

### 3.6. IMU Calibration and Validation

To evaluate the accuracy of the Wi-Fi smart particle IMU, a controlled pendulum experiment was performed prior to analysing motion measurements obtained inside the rotating drum. Pendulum-based experiments are commonly used for validating inertial measurement units because the motion of the sensor can be independently measured and compared with IMU-derived signals [36,37,38,39]. The experiment therefore provides an external reference for assessing IMU accuracy and drift before applying the sensor in environments where direct ground-truth measurements are not available.

#### 3.6.1. Pendulum Experimental Setup and Ground Truth Acquisition

The pendulum experiment was designed to generate repeatable oscillatory motion that could be independently measured using video tracking. The WitMotion Wi-Fi sensor was suspended using a string with a length of 30 cm measured from the pivot to the centre of the sensor. The suspended sensor acted as the pendulum bob and was allowed to oscillate freely within a vertical plane. The pendulum was released from an initial angle of approximately 30 degrees, which produced repeatable oscillations that gradually decayed due to damping caused by air resistance and pivot friction. During each trial, the IMU recorded triaxial acceleration and angular velocity while the pendulum oscillated. The recorded IMU data were transmitted and logged for later analysis.

To obtain ground-truth motion, the pendulum experiment was simultaneously recorded using an external camera. The pendulum motion was analysed using Tracker video analysis software (6.2.0), a free open-source tool developed within the Open-Source Physics framework that allows the extraction of position, velocity, and acceleration data from recorded motion videos [40,41]. The position of the pendulum sensor was tracked frame by frame, which allowed extraction of the pendulum trajectory and angular displacement as a function of time. From the tracked trajectory, the linear velocity and angular velocity of the pendulum were computed and used as ground-truth reference signals.

**Figure 5:**
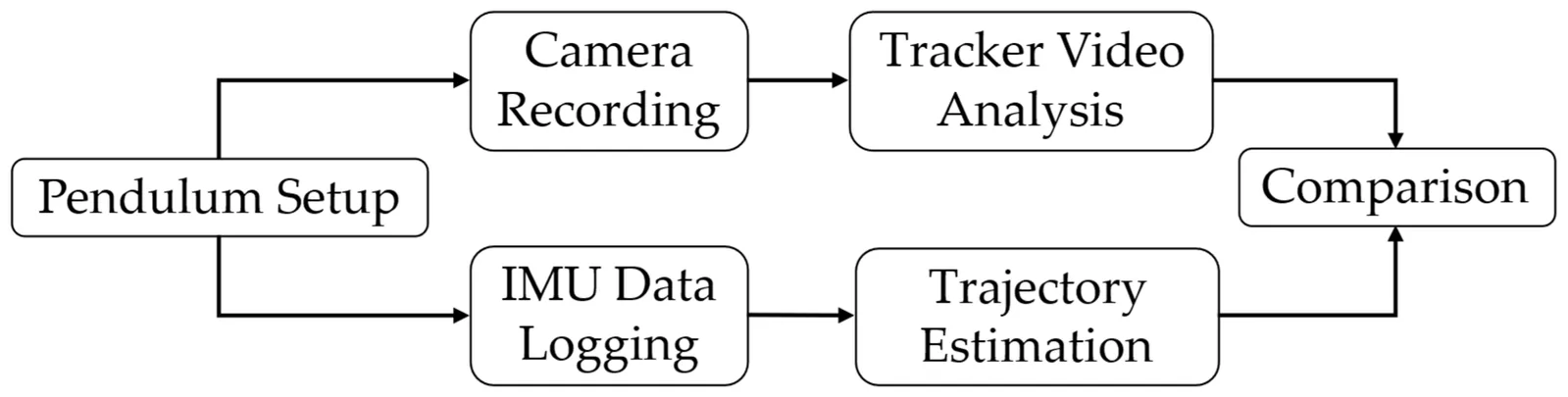
Workflow of the pendulum experiment and motion extraction using Tracker software.

Figure 5 illustrates the workflow of the pendulum experiment, including sensor mounting, video acquisition, and motion extraction.

**Figure 6:**
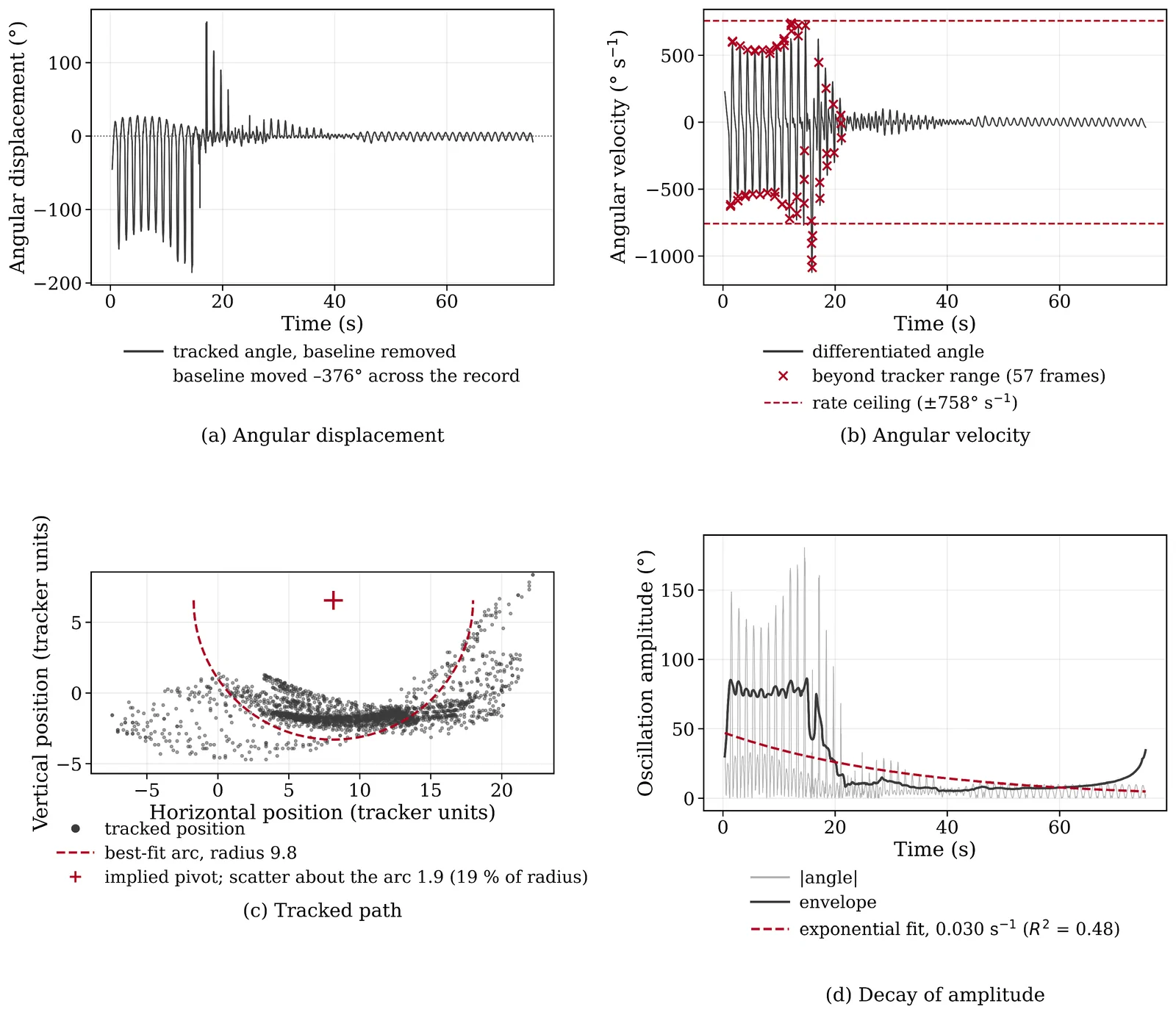
Pendulum motion obtained from video tracking. (**a**) Angular displacement. (**b**) Angular velocity. (**c**) Reconstructed two-dimensional motion. (**d**) Decay of oscillation amplitude.

The angular displacement and velocity signals show periodic oscillations typical of pendulum dynamics, while the reconstructed trajectory confirms motion along a circular arc about a fixed pivot. The gradual reduction in amplitude indicates damping during the experiment. This is shown in Figure 6.

#### 3.6.2. Axis Selection and Temporal Alignment

As the IMU records motion in the local sensor coordinate frame, the axis corresponding to the pendulum swing direction must first be identified before comparison with the ground-truth measurements. The swing axis is identified by scoring each gyroscope axis against the optical reference. Every axis is cross-correlated with the reference overlapping windows, and the axis with the highest median correlation is selected. On this record, the gyroscope Z-axis attains a median |r| of 0.88, against 0.46 for the next best, and is used throughout.

Since the IMU and the video tracking system operate with independent clocks, the two signals were temporally aligned prior to comparison. The ground-truth motion was obtained using the Tracker video analysis software, which extracts position and kinematic quantities from recorded motion sequences [38,39]. From the tracked pendulum trajectory, the angular displacement θ(t) was obtained, and the corresponding angular velocity was calculated as

$$\omega_{GT}(t)=\frac{d\theta (t)}{dt}$$

The IMU gyroscope measurement, $\omega_{IMU}(t)$, was then aligned with the ground-truth angular velocity using cross-correlation to estimate the relative time offset between the two signals

$$\Delta t=arg\underset{\tau }{max}\int \omega_{IMU}(t)\;\omega_{GT}(t+\tau )\;dt$$

where τ represents the candidate time shift. The inertial unit samples at 100 Hz and the video at 30.30 Hz on independent, free-running clocks. The offset between them is therefore estimated locally, in overlapping 12 s windows, rather than assumed to be constant over the record; the median magnitude of the local offset is 0.99 s. Alignment is computed in discrete form as

(5) $$R\left[m\right]=\sum_{n}\omega_{IMU}\left[n\right]\;\omega_{ref}\left[n+m\right],\;\;\;\;\;\;\Delta t=\frac{m^{*}}{f_{s}}$$

with both series placed on the video sampling grid, so that the lag index maps to a known time step and the alignment resolution is one grid sample.

The tracked angle is unwrapped before differentiation, since the raw angle wraps at ±180° and differentiating a wrapped signal introduces steps of ±360° per frame. Angular velocity is obtained using Savitzky–Golay differentiation with a window of 21 samples (0.693 s) and polynomial order 3. For a per-frame angular tracking uncertainty of $\sigma_{\theta }$ = 0.50°, the uncertainty propagated through the differentiating kernel is

(6) $$\sigma_{\omega }=\sigma_{\theta }\;\left\|g\right\|$$

where g is the Savitzky–Golay derivative kernel, giving $\sigma_{\omega }$ = 1.38°/s. This is small relative to the angular velocities of interest, so optical noise is not the factor limiting the comparison.

The optical reference is not uniformly valid across the record. A frame in which the tracked angle advances by more than 25° records a bob displacement larger than the track can follow between exposures, and the velocity inferred from it is an artefact rather than a measurement. At the measured frame rate, this corresponds to an angular rate ceiling of 758°/s. In total, 57 frames (2.5%) exceed the threshold, with the largest step being 173°, and these frames are excluded. The reference remains within its resolvable range continuously from 28 s onward.

#### 3.6.3. IMU Signal Processing and Validation Against Ground Truth

**Figure 7:**
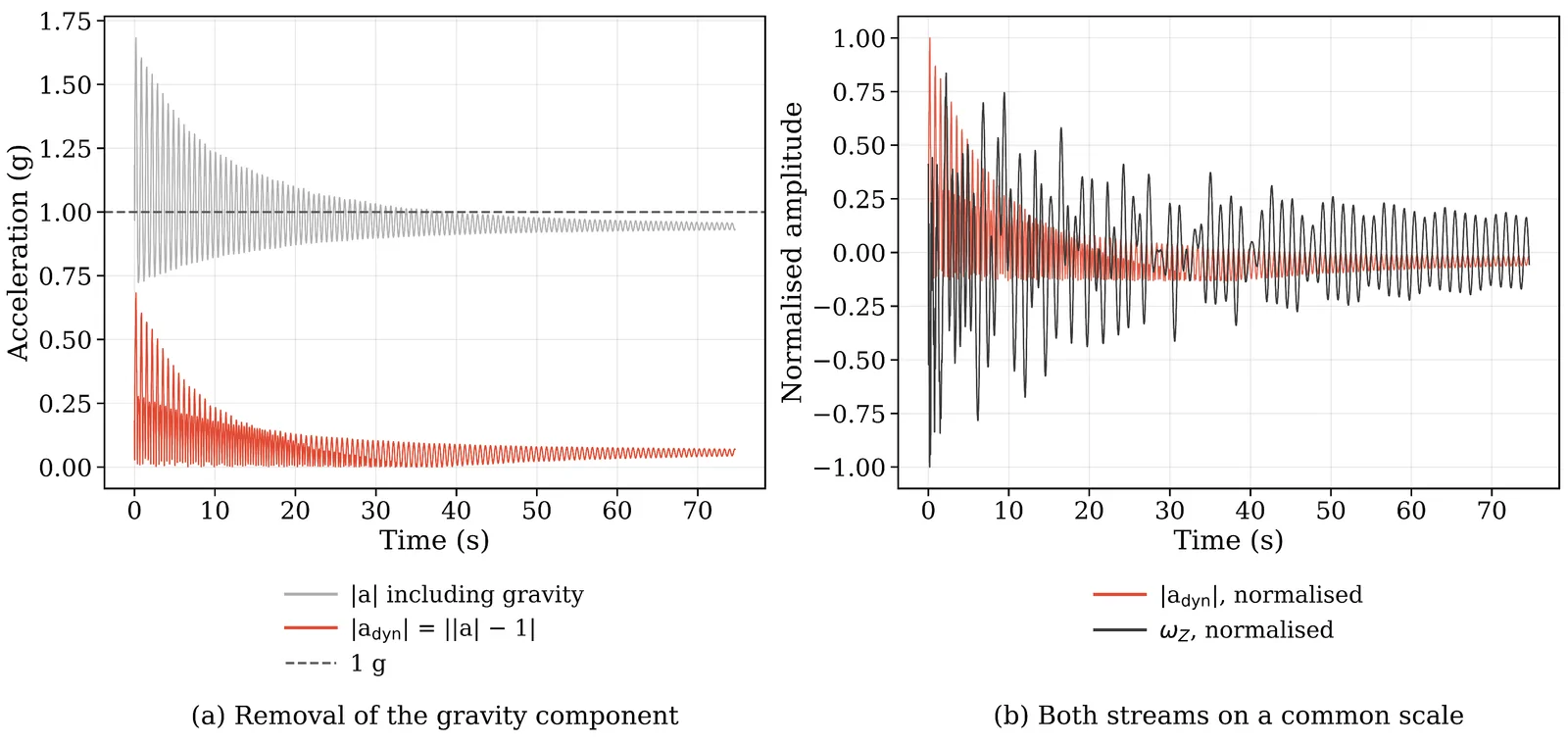
Inertial signal preprocessing. (**a**) Raw acceleration magnitude and gravity-removed dynamic acceleration. (**b**) Normalised dynamic acceleration and angular velocity.

Raw IMU measurements contain sensor bias, gravitational acceleration components, and measurement noise, which were addressed before meaningful motion analysis could be performed. In this study, a preprocessing pipeline was applied to both accelerometer and gyroscope measurements to obtain physically meaningful signals for validating pendulum motion. The accelerometer signal was first analysed through the magnitude of the 3-axis acceleration vector. The raw acceleration magnitude includes both the dynamic acceleration generated by pendulum motion and the constant gravitational component. To isolate the acceleration related to motion, the gravitational component was removed to obtain the dynamic acceleration magnitude. Figure 7a shows the raw acceleration magnitude together with the gravity-removed dynamic acceleration signal. The resulting dynamic acceleration clearly captures the oscillatory behaviour of the pendulum motion, while the gradual decay of the amplitude reflects the damping of the oscillation over time.

To verify the consistency between accelerometer and gyroscope measurements, a qualitative comparison was performed between the dynamic acceleration magnitude and the angular velocity recorded by the selected gyroscope axis. For comparison, the two signals were normalised to highlight their oscillatory structure. As shown in Figure 7b, the normalised acceleration magnitude and the gyroscope angular velocity exhibit similar periodic behaviour and frequency characteristics, indicating that both sensors capture the same underlying pendulum dynamics. This agreement confirms the reliability of the IMU measurements after preprocessing.

**Figure 8:**
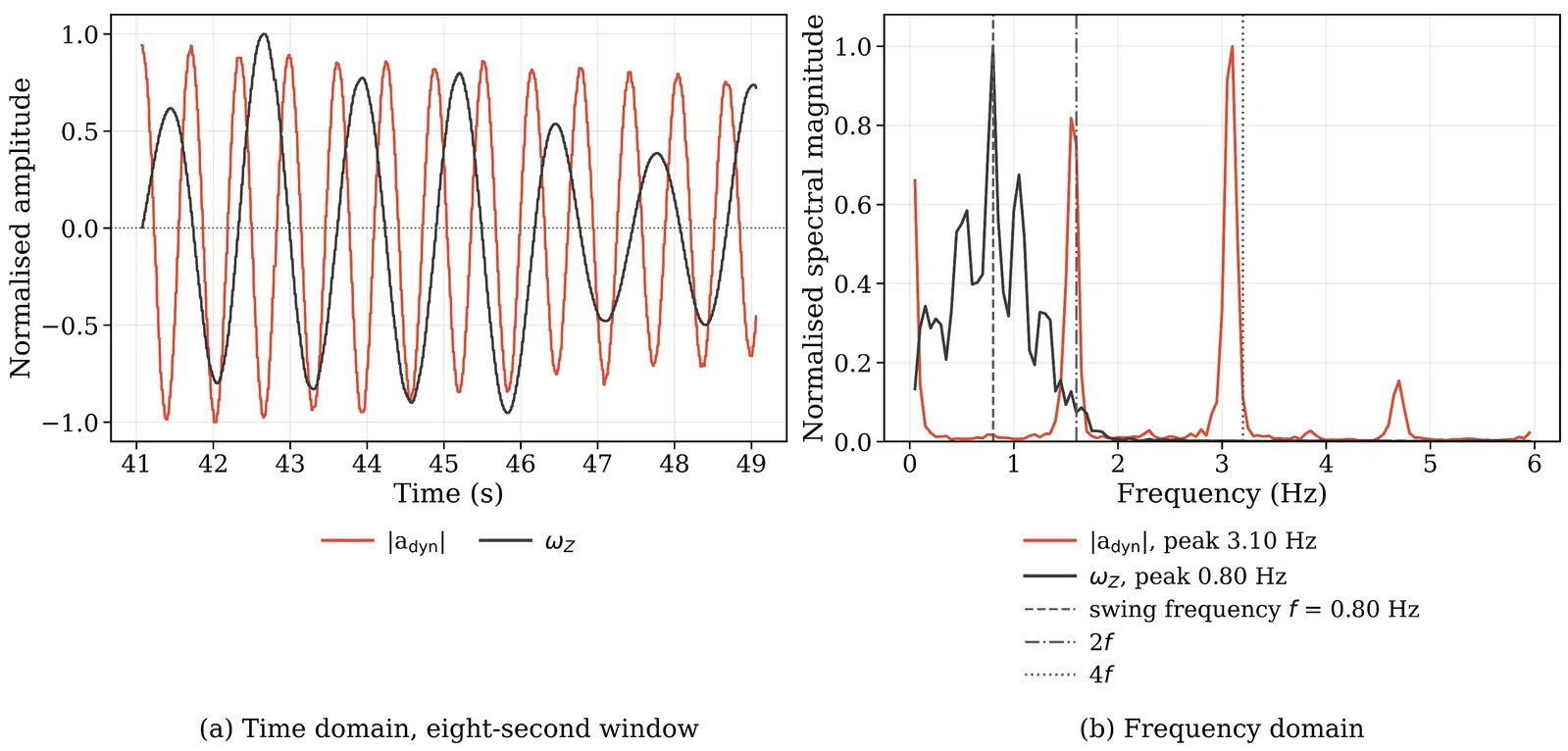
Consistency between accelerometer and gyroscope measurements. (**a**) Time domain. (**b**) Frequency domain.

To further validate the consistency of the IMU measurements, both time domain and frequency domain analyses were performed. In the time domain, the normalised dynamic acceleration and gyroscope-derived angular velocity (ω) shown in Figure 8a exhibit similar oscillatory behaviour, with a gradual decay in amplitude reflecting the damping characteristics of the pendulum motion. Although the signal shapes differ due to the distinct sensing principles, the periodic structure and oscillation frequency remain consistent across both measurements.

In the frequency domain, the fast Fourier transform (FFT) was applied to both signals to identify their dominant oscillation components. As illustrated in Figure 8b, both spectra exhibit clear dominant low-frequency peaks corresponding to the fundamental pendulum oscillation, while higher-frequency components are primarily associated with sensor noise and minor disturbances. The close agreement in dominant frequency between the two signals confirms that the accelerometer and gyroscope consistently capture the same underlying motion dynamics.

**Figure 9:**
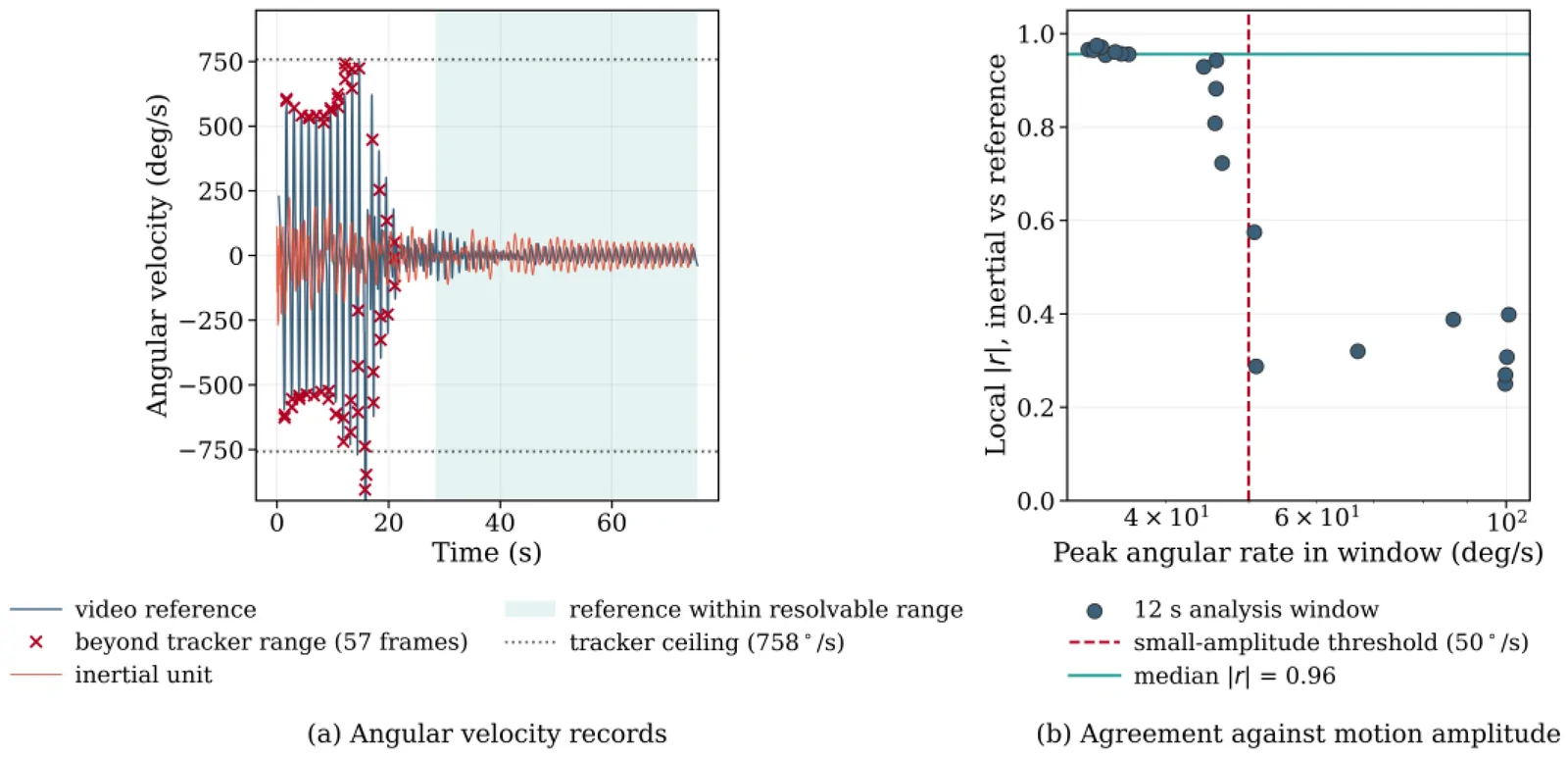
Validation of the smart particle inertial unit against video tracking. (**a**) Angular velocity from both instruments, with the interval over which the optical reference remains within its resolvable range shaded, and out-of-range frames marked. (**b**) Local correlation between the two records against the peak angular rate in each analysis window.

After preprocessing, the IMU measurements were compared with the ground-truth motion obtained from Tracker video analysis. The comparison focused on the angular velocity signals derived from the two measurement sources. Agreement between the two instruments is quantified as the distribution of local correlation over the analysis windows and is reported separately for the two motion regimes the experiment spans. Below 50°/s, the pendulum is effectively isochronous, and the optical reference is well within its resolvable range; across the 13 windows in this regime, the median |r| is 0.96, with a minimum of 0.72. Above that rate, the median falls to 0.31. The degradation has two identifiable causes, neither of which implies disagreement between the instruments: the optical reference approaches its rate ceiling, and a pendulum released to a large angle is not isochronous, so its period changes measurably within a correlation window. Validation is therefore established in the small-amplitude regime, and the large-amplitude regime is reported as a limitation of the optical reference. Figure 9 shows both.

Both signals capture the periodic oscillatory behaviour of the pendulum motion and exhibit a gradual reduction in amplitude over time, consistent with the damping behaviour of the system. Differences between the signals are more pronounced during the initial oscillations, where the angular velocity values are highest and the motion changes rapidly.

To quantify the discrepancy between the two signals, the instantaneous angular velocity error was calculated as

$$e(t)=\omega_{IMU}(t)-\omega_{GT}(t)$$

where $\omega_{IMU}(t)$ represents the IMU gyroscope measurement and $\omega_{GT}(t)$ denotes the angular velocity derived from the tracker measurements. The natural frequency recovered from the inertial record, 0.800 Hz, agrees with the optically derived value of 0.850 Hz to 5.9%. Together with the local correlation in the small-amplitude regime, this establishes that the smart particle inertial unit resolves oscillatory motion faithfully and supports its use inside the drum where an independent optical reference is not available.

### 3.7. Smart Particle Motion Analysis

The motion of the smart particle inside the rotating drum influences both the dynamic behaviour of the particle and the wireless communication performance observed in the previous section. As the drum rotates, the particle is carried up the shell by the lifters, released, and falls back onto the wall, so its motion consists of repeated lifting, free flight, and impact. These motion phases influence the orientation and position of the smart particle and consequently affect the wireless communication behaviour of the embedded sensor. Prior to analysis, the IMU signals were processed using the calibration and filtering procedures described in Section 3.6 to remove sensor bias noise and drift. The resulting calibrated IMU measurements provide a reliable representation of the particle motion inside the rotating drum. To analyse these dynamics, the inertial measurement unit data recorded by the WitMotion Wi-Fi sensor were examined under stationary conditions and at different drum rotational speeds, including 16 RPM, 18 RPM, and 20 RPM. The motion analysis was performed in the time and frequency domains. To quantify the influence of rotational speed on particle motion, statistical features were extracted from the IMU measurements, including the root mean square (RMS), the 95th percentile (P95), and peak values of both dynamic acceleration and angular velocity. These metrics provide complementary information on sustained motion intensity, high-amplitude behaviour, and extreme transient events.

## 4. Results

### 4.1. RSSI Behaviour

This section presents the analysis of received signal strength indicator measurements obtained from the smart particle sensors under stationary and rotating operating conditions. The objective of this analysis is to understand how wireless communication performance is influenced by sensor motion and by the metallic enclosure of the rotating drum.

**Figure 10:**
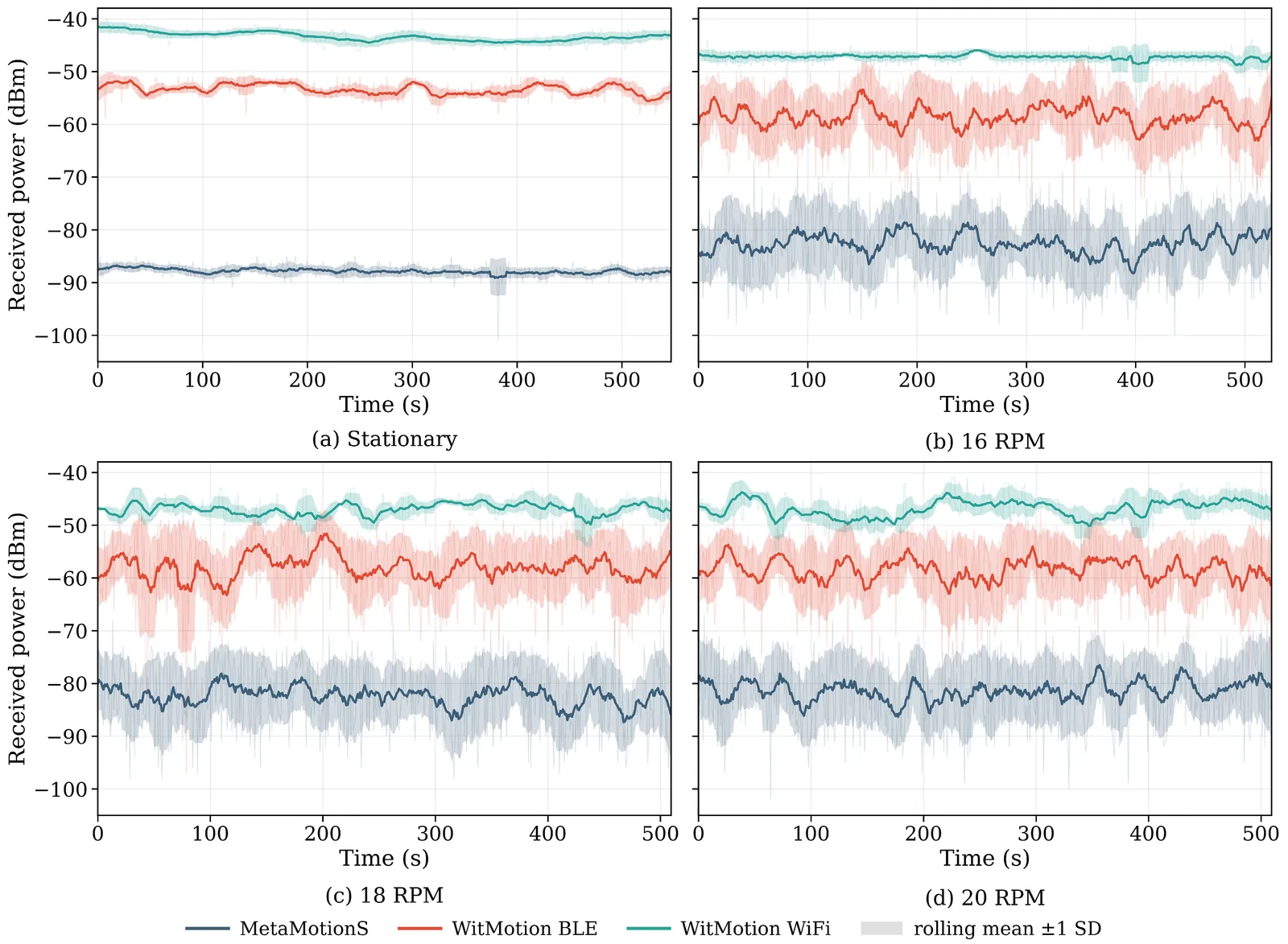
Received power under each condition, showing raw samples, a centred rolling mean, a ±1 SD band, and the 95% confidence interval of the record mean at the right-hand edge, with *n* and effective sample size annotated. Each panel is shown over the common interval covered by all three particles. (**a**) Stationary. (**b**) 16 RPM. (**c**) 18 RPM. (**d**) 20 RPM.

Figure 10 illustrates the RSSI time-series behaviour of the MetaMotion BLE, WitMotion BLE, and WitMotion Wi-Fi sensors across stationary and rotating conditions. Under stationary conditions in Figure 10a, all sensors exhibit relatively stable RSSI values with minimal fluctuations, establishing a baseline for communication performance. The WitMotion Wi-Fi sensor records the strongest signal levels, followed by the WitMotion BLE sensor, while the MetaMotion BLE sensor exhibits significantly weaker signal strength. These differences arise due to variations in transmission power, antenna design, and the underlying communication protocols.

Descriptive statistics with uncertainty are given in Table 5. Because received-power samples in a rotating system may be serially correlated, an effective sample size is estimated for every record as

(7) $$n_{eff}=\frac{n}{\tau },\;\;\;\;\;\;\;\;\tau =1+2\sum_{k=1}^{K}\rho_{k}$$

where $\rho_{k}$ is the sample autocorrelation at lag *k*, and *K* is chosen by the initial-positive-sequence rule. Confidence intervals are computed both from the t distribution with n replaced by *n*_eff_ and by a moving-block bootstrap with 10,000 resamples. Under rotation, the integrated autocorrelation time is close to unity (τ = 1.07–13.14), so the samples are close to independent. Under stationary conditions, they are not: τ reaches 30.9 and *n*_eff_ falls to 16, because a stationary link drifts slowly rather than fluctuating from sample to sample.

When the drum begins to rotate, the RSSI signals exhibit increased variability (Figure 10b–d) due to continuous changes in sensor position, antenna orientation, and signal propagation paths within the metallic drum. Multipath effects and partial signal obstruction further contribute to fluctuations in RSSI. As rotational speed increases, the magnitude of these fluctuations becomes more pronounced, particularly for the BLE-based sensors. The MetaMotion BLE sensor demonstrates the highest variability, while the WitMotion BLE sensor shows moderate fluctuations. In contrast, the WitMotion Wi-Fi sensor maintains comparatively stable signal levels with smaller deviations across all operating conditions.

Differences between particles are statistically significant in every condition (Welch heteroscedastic ANOVA, *p* < 0.001), with Kruskal–Wallis in agreement. Welch’s test is used in preference to classical ANOVA because the group variances differ by more than an order of magnitude. Games–Howell post hoc comparisons, which assume neither equal variances nor equal group sizes, return *p* < 0.001 for all pairwise comparisons with |Cliff’s δ| > 0.96, indicating distributions that barely overlap.

Fade behaviour is quantified relative to a running local median over 31 samples, so that each particle is referenced to its own baseline and the spread in absolute level does not distort the comparison. A deep fade is defined as a sample more than two robust standard deviations below that baseline. The deep-fade rate of the Wi-Fi particle rises from 0.80% at rest to 11.80% at 20 RPM, while its fade depths remain small, a speed dependence that is not visible in the record-level standard deviation.

### 4.2. Sensor Performance Evaluation

**Figure 11:**
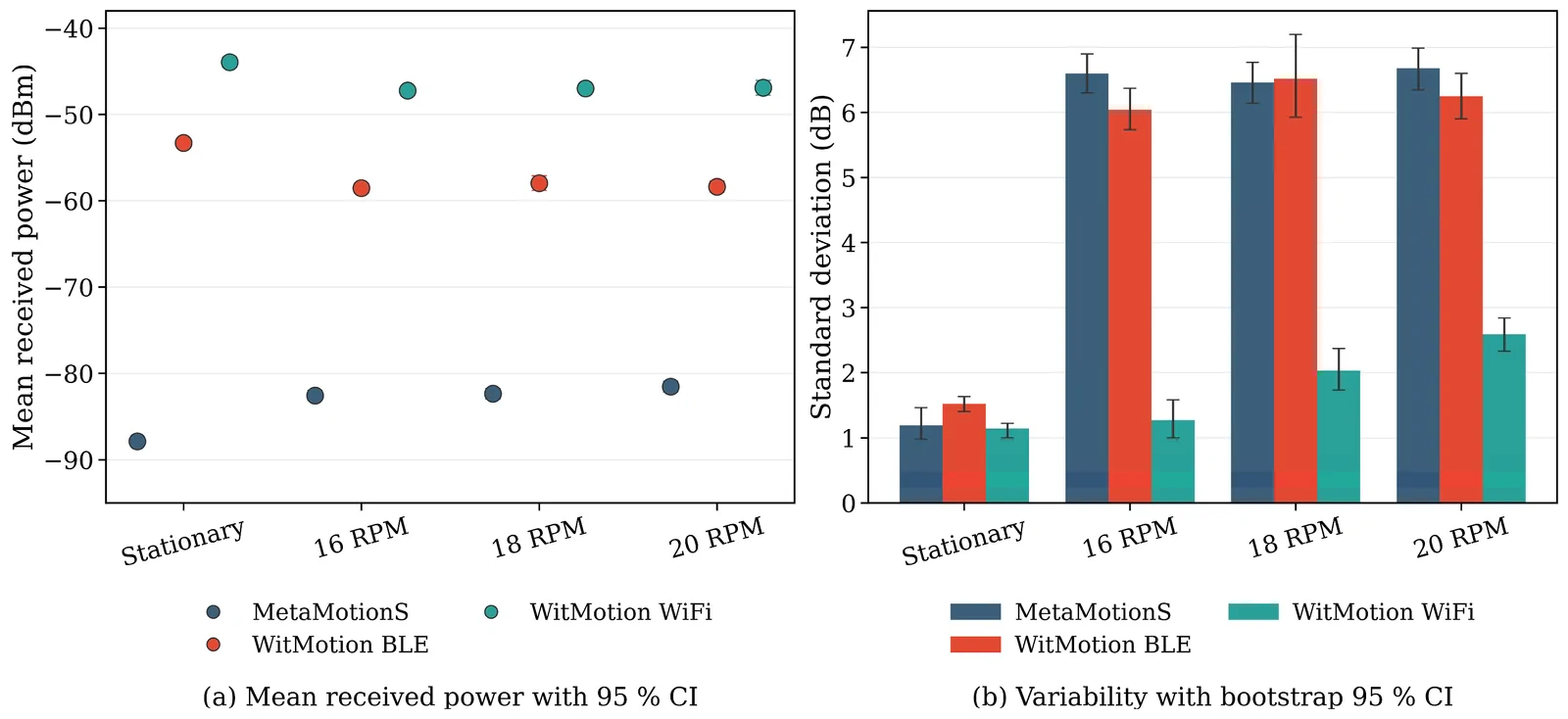
(**a**) Mean received power with 95% confidence interval. (**b**) Standard deviation with bootstrap 95% confidence interval.

The proposed sensor selection framework was applied to the experimental RSSI data to evaluate and compare the performance of the smart particle sensors. Figure 11 presents mean received power with its 95% confidence interval, and the standard deviation with its bootstrap confidence interval. An interval is shown on the standard deviation, as well as on the mean, because the central claim concerns variability, so the uncertainty on the variability estimate is the one that matters. The WitMotion Wi-Fi sensor consistently exhibits higher mean RSSI values compared to the BLE sensors under both stationary and rotating conditions. The MetaMotion BLE sensor shows the lowest signal strength, while the WitMotion BLE sensor demonstrates intermediate performance.

The standard deviation results indicate that the Wi-Fi sensor maintains lower RSSI variability across all operating conditions. In contrast, the BLE-based sensors exhibit higher variability, with fluctuations increasing under rotational motion. Averaged over the rotating conditions, the standard deviations are 1.96 dB for the Wi-Fi particle and 6.27 and 6.58 dB for the two BLE particles.

#### 4.2.1. Link-Budget Normalisation

**Figure 12:**
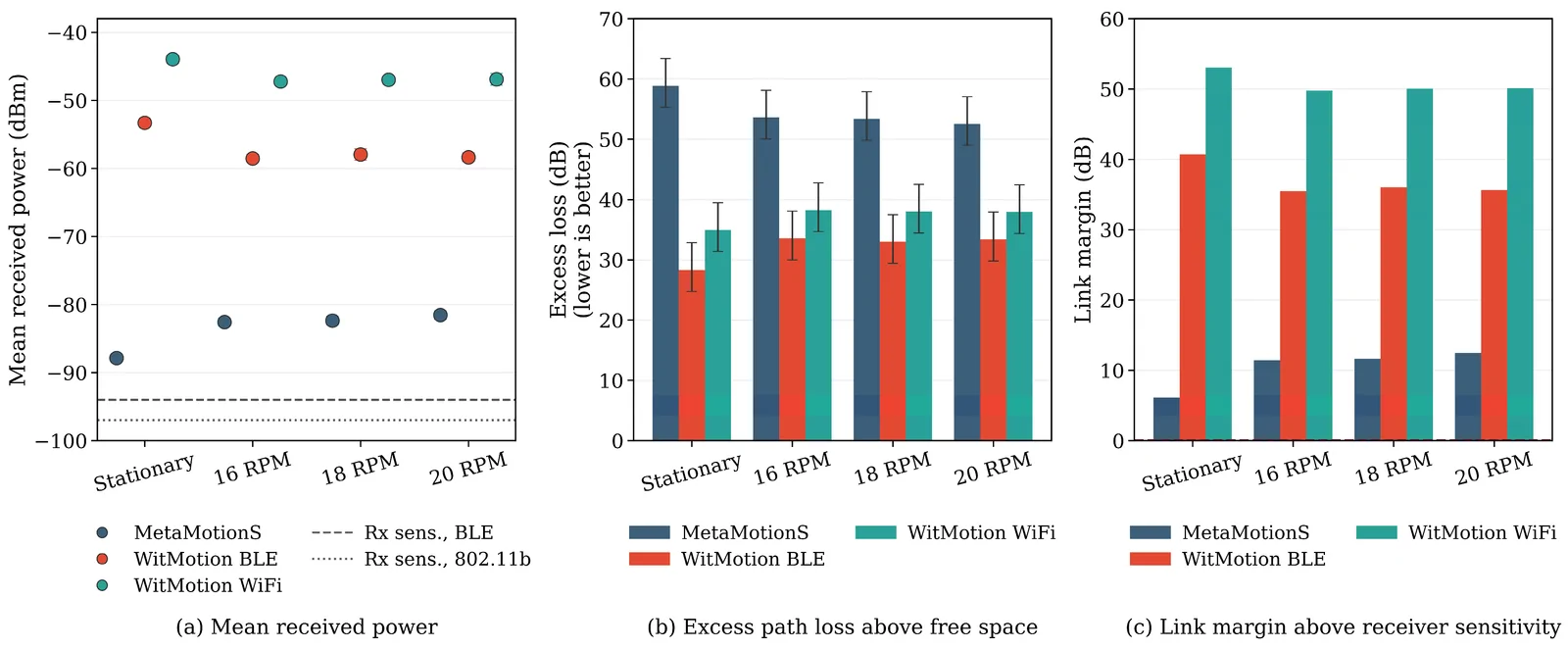
Link-budget normalisation. (**a**) Mean received power. (**b**) Excess path loss above free space after subtracting each device’s transmit power. (**c**) Link margin above receiver sensitivity.

Received power conflates the transmission power of a device with the treatment the channel gives it. To separate the two, the comparison is expressed as excess path loss above free space,

(8) $$L_{ex}=\underset{\mathrm{total}\;\mathrm{path}\;\mathrm{loss}}{\underbrace{P_{EIRP}-\mu_{RSSI}}}-L_{FS},\;\;\;\;\;\;\;L_{FS}=20{log}_{10}\left(\frac{4\pi df}{c}\right)$$

where *d* is the particle-to-receiver distance, f is the carrier frequency, and c is the speed of light. At the mid-depth distance of 275 mm and 2.44 GHz, the free-space reference is 29.0 dB, varying between 24.5 and 32.6 dB over the axial extent of the drum. Figure 12 shows the raw levels, the excess loss, and the resulting link margins. Figure 12a shows the mean received power with 95% confidence intervals, shown as markers because the decibel-milliwatt scale has no true zero; the dashed and dotted lines mark the receiver sensitivities for BLE and 802.11b. Figure 12b shows the excess path loss above free space after subtracting each device’s transmit power, referenced to the mid-depth particle-to-receiver distance; the error bars give the variation arising from axial position within the drum. Lower values indicate a more efficient link. Figure 12c shows the link margin that measures the reliability and robustness of the wireless link.

All three links close on the mean. Referenced to the manufacturers’ receiver sensitivities, the mean margins under rotation are 11.4–12.5 dB for MetaMotionS, 35.5–36.0 dB for the WitMotion BLE particle, and 49.8–50.1 dB for the Wi-Fi particle. Feasibility is therefore not in question at this scale: a sealed 2.4 GHz radio inside a rotating metallic drum sustains a link in every configuration tested. The ordering is not set by transmit power alone, as MetaMotionS and the WitMotion BLE particle are separated by 24 dB of margin across 4 dB of transmit power at most.

The mean level does scale with transmit power, and the comparison between the Wi-Fi and MetaMotionS particles reflects that: their separation under rotation is 35.1 dB, of which at most 20.5 dB is transmit power, leaving at least 14.6 dB arising elsewhere.

The comparison between the two BLE particles is more informative because they share a protocol class, a frequency band, a rig, and a receiver, and differ by 23.9 dB. Neither manufacturer publishes a configured transmit power, so rather than assume one, the comparison is bounded over the documented range of each radio: the Nordic nRF52840 operates between 0 and +8 dBm and the Nordic nRF52832 between 0 and +4 dBm. Since MetaMotionS is the weaker of the two, the configuration most favourable to a transmit-power explanation is MetaMotionS at its minimum and WitMotion BLE at its maximum, a difference of 4 dB. At least 19.9 dB of the measured separation therefore cannot be attributed to transmit power under any admissible configuration, and must arise from the device implementation: antenna, enclosure, placement, and firmware. The conclusion does not depend on the exact settings being known.

**Figure 13:**
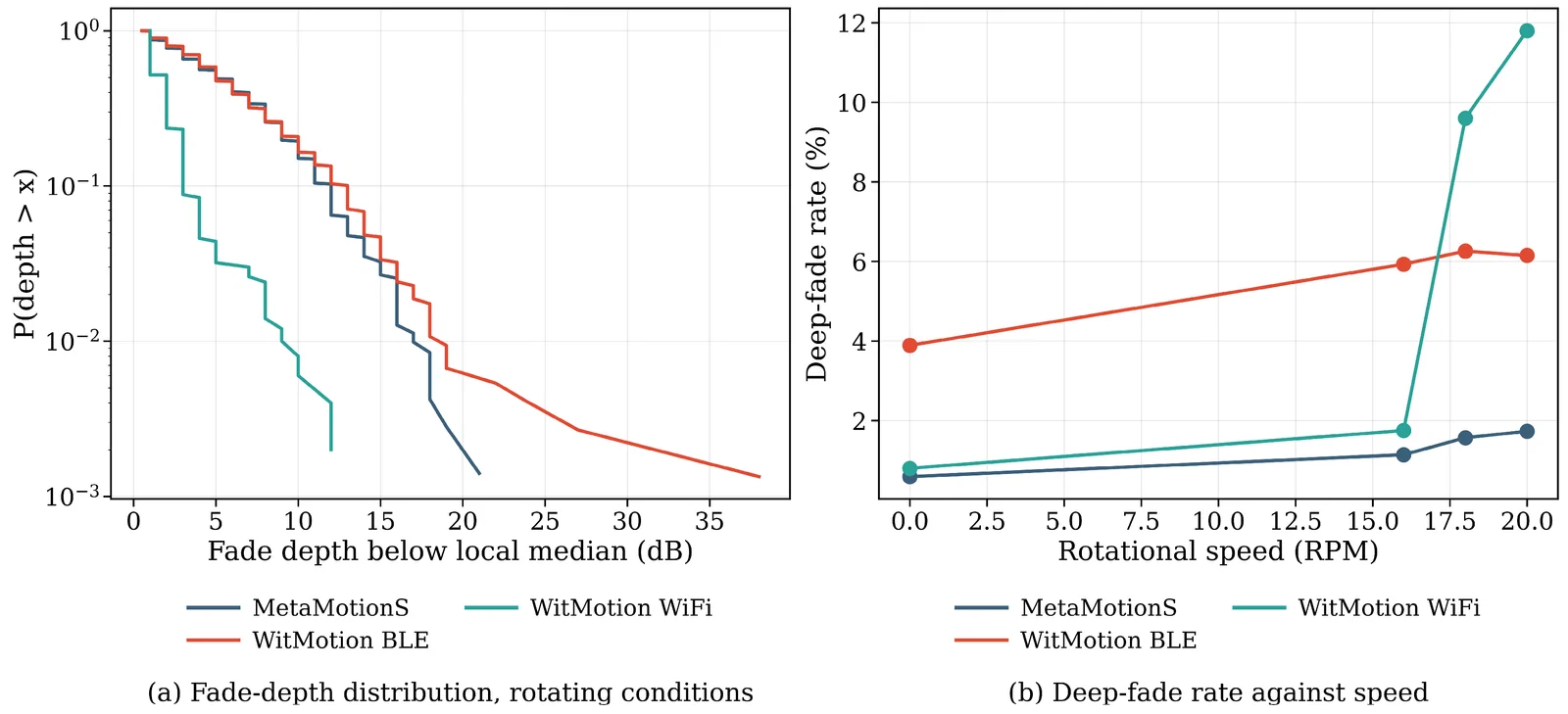
Fade statistics referred to a local median baseline. (**a**) Complementary cumulative distribution of fade depth, rotating conditions pooled. (**b**) Deep-fade rate against rotational speed.

A second line of evidence follows from the simultaneous logging of the two WitMotion particles. Their samples are paired observations of one channel realisation by two radios. The fluctuations were driven by the shared environment, such as drum geometry, rotation, and receiver placement. The two series would rise and fall together. They do not: the correlation between their fluctuations about their own means is +0.064 at 16 RPM, −0.098 at 18 RPM, and −0.083 at 20 RPM. The fading is device-specific, as shown in Figure 13.

A margin computed on the mean is not sufficient to establish reliability, because whether a packet is received depends on the instantaneous level and not on its average. Setting the margins of Figure 12c against the fade depths of Figure 13 shows how much of each margin the fading consumes. The Wi-Fi particle fades by at most 12 dB and so retains 38 dB at its deepest observed excursion. The WitMotion BLE particle fades by up to 38 dB against a mean margin of 36 dB, so a single worst-case excursion consumes its entire budget; it operates reliably because such fades are rare, not because the budget accommodates them. For MetaMotionS, the mean margin of 11.4–12.5 dB is exceeded by fades of 18–21 dB, and 2.1–3.6% of its rotating samples fall below the receiver sensitivity outright, rising to 8.5–10.6% if a 3 dB implementation reserve is required; its minimum recorded level of −102 dBm lies 8 dB below sensitivity. A positive mean margin therefore does not by itself qualify a candidate, and the reserve required above it is set by the fade distribution of the individual device rather than by a general rule.

**Figure 14:**
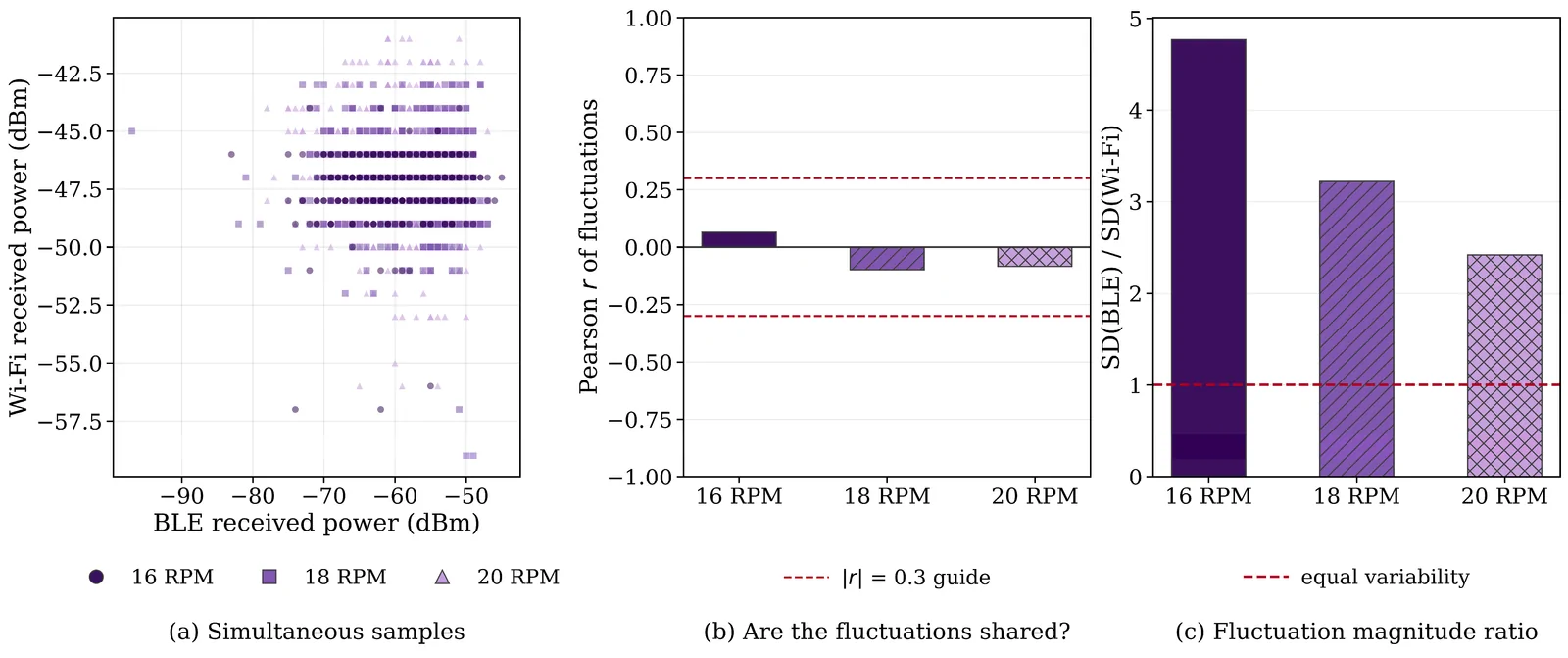
Paired comparison of two particles logged simultaneously through a single receiver. (**a**) Simultaneous received-power samples. (**b**) Correlation of the fluctuations about each device’s own mean, by speed. (**c**) Ratio of fluctuation magnitudes.

Figure 14 presents the comparison, and the ratio of fluctuation magnitudes between the two devices, 2.4–4.8, is a second-order quantity that is largely independent of transmit power for a receiver operating above its noise floor.

MetaMotionS operates 11.8 dB above the noise floor assumed here, so a portion of its apparent variability may be receiver-limited rather than channel-limited, and its variability figure should be read with that in mind. Two further considerations bound these margins, and both act in the same direction. The sensitivity figures are manufacturers’ published values, and no reference transmitter calibration was performed, so each margin carries the systematic offset discussed in Section 3.3; at approximately 5 dB, this is immaterial against 50 dB and material against 12 dB. The reference level is the receiver sensitivity rather than a measured ambient noise floor, so these are upper bounds on the margin available where the 2.4 GHz band is occupied. Both qualifications act equally on every device in decibels, but not equally in consequence, because MetaMotionS has the least margin to surrender. An ambient noise floor 9 dB above the assumed level, for instance, would leave the Wi-Fi particle at 38 dB and the WitMotion BLE particle at 27 dB, while reducing MetaMotionS to 3.5 dB. The same 5 dB calibration uncertainty is a tenth of the Wi-Fi particle’s margin and over a third of MetaMotionS’s.

#### 4.2.2. Endurance and Energy per Packet Delivered

Applying Equation (2) with the manufacturers’ published capacities and operating currents, and the delivered packet rates measured here, the Wi-Fi particle runs for 3.0 h against 10.0 h for both BLE particles. Expressed as energy per successfully delivered packet, the Wi-Fi particle costs 302 mJ, against 94 mJ for the WitMotion BLE particle and 38 mJ for MetaMotionS, a factor of 8.0 between the extremes. The particle with the most stable link is therefore also the most expensive to operate, by a wide margin. Values are given in Table 6.

#### 4.2.3. Multi-Criteria Evaluation

**Figure 15:**
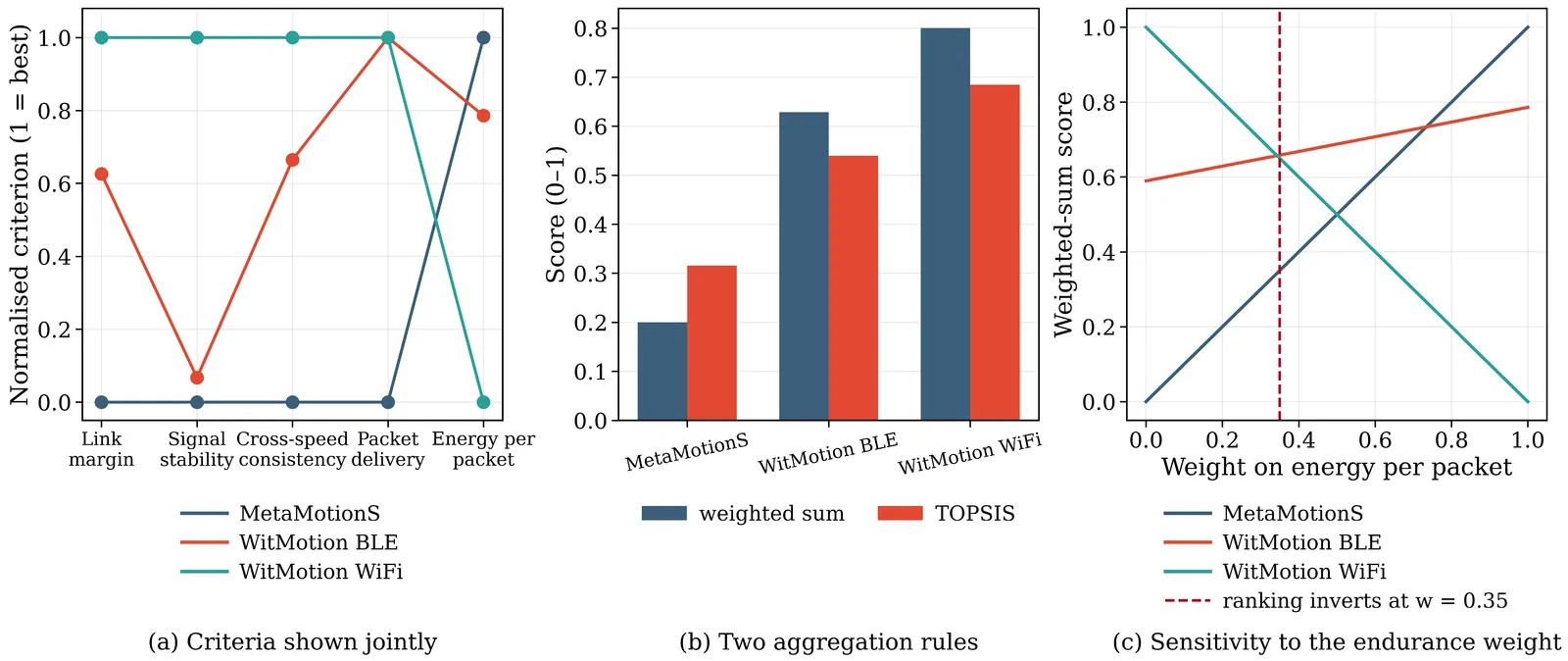
Multi-criteria evaluation. (**a**) The five normalised criteria shown jointly. (**b**) Weighted-sum and TOPSIS scores. (**c**) Sensitivity of the ranking to the weight placed on energy per delivered packet, with the crossing marked. Min–max normalisation across three candidates necessarily places the weakest at zero on each criterion.

Applying the framework of Section 3.5 to the rotating conditions ranks the WitMotion WiFi particle first under both aggregation rules, with a weighted-sum score of 0.800 and a TOPSIS index of 0.684; the two rules agree on the full ordering. Sweeping the weight on signal strength against the weight on variability across its entire range leaves the ordering unchanged, so that trade-off does not arise for this candidate set. Figure 15 shows the criteria jointly rather than collapsed into a single index.

Endurance does change the answer. Because the Wi-Fi particle is best on every link-quality criterion and worst on energy per delivered packet, a crossing must exist, and it lies at an energy weight of 0.35: below it, the WitMotion WiFi particle ranks first and above it is the WitMotion BLE particle. An application required to run for days between charges should therefore not select on link quality alone. Identifying where the preference inverts is more useful than asserting a single winner, and it directly answers the question of whether absolute signal strength or stability should be prioritised: above a satisfied link margin, neither dominates, and endurance decides.

### 4.3. Smart Particle Motion Analysis in the Rotating Drum

The motion of the smart particle inside the rotating drum was analysed based on time and frequency domains as illustrated in the subsequent subsections. The time-domain characteristics of the smart particle motion provide direct insight into its dynamic behaviour within the rotating drum.

Table 7 shows the motion statistics for different runs. The time durations and gyro saturation percentages are presented.

#### 4.3.1. Time-Domain Motion Behaviour

**Figure 16:**
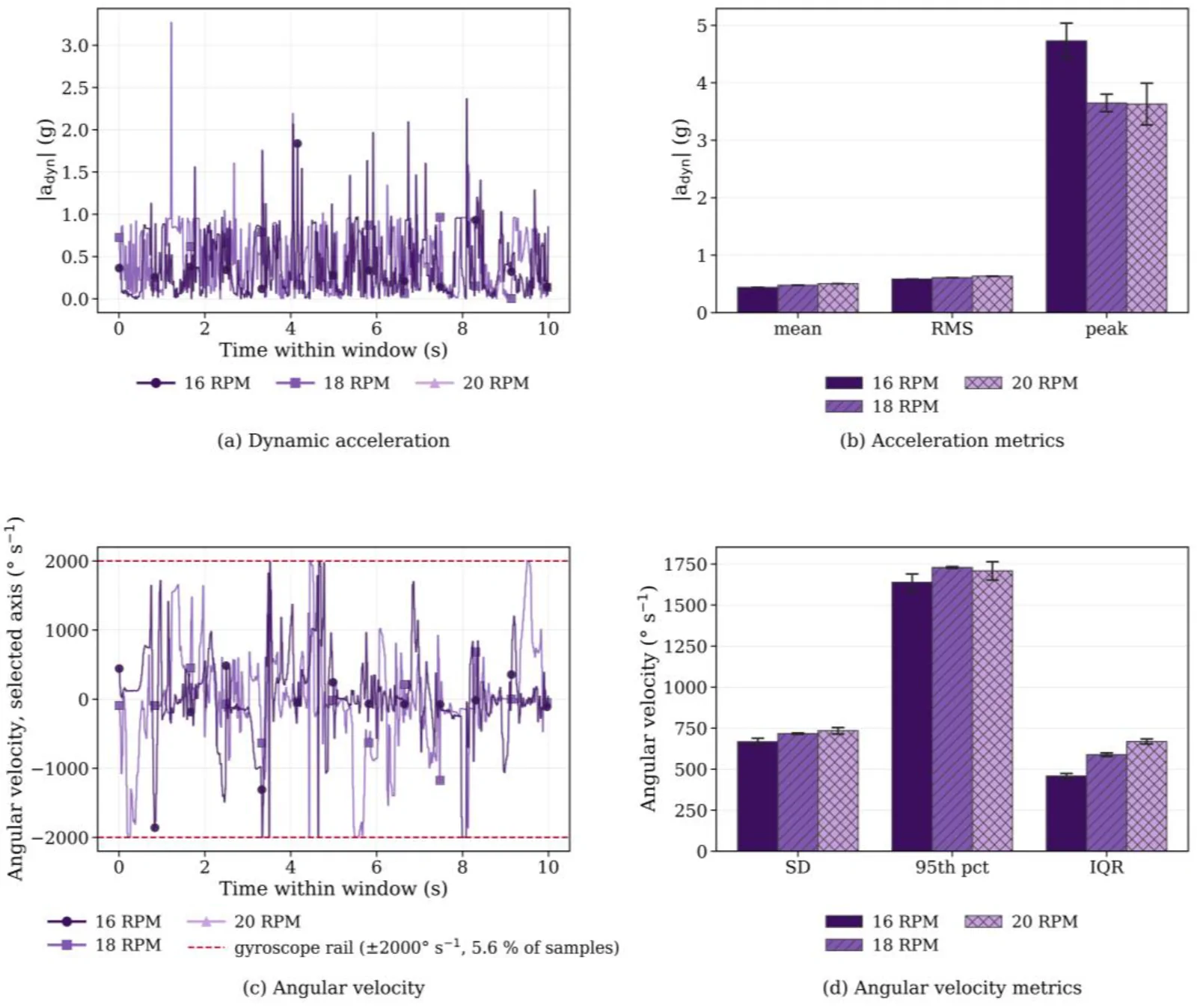
Time-domain motion behaviour of the smart particle under varying rotational speeds. (**a**) Dynamic acceleration magnitude, (**b**) acceleration metrics including mean, RMS, and peak values, (**c**) angular velocity along the selected gyroscope axis, and (**d**) gyroscope metrics, including standard deviation, 95th percentile, and interquartile range for 16 rpm, 18 rpm, and 20 rpm conditions.

Figure 16 presents the dynamic acceleration magnitude and angular velocity responses of the particle at rotational speeds of 16 RPM, 18 RPM, and 20 RPM, along with their corresponding summary statistics.

The dynamic acceleration signals shown in Figure 16a exhibit stochastic fluctuations arising from repeated lifting, rolling, and impact events between the particle and the drum wall. At 16 rpm, the acceleration profile remains relatively stable with moderate amplitude variations, indicating controlled motion dominated by periodic lifting and rolling. As the rotational speed increases to 18 rpm, the signal becomes more irregular with increased dispersion, reflecting higher particle mobility and more frequent impacts with the drum boundary. At 20 rpm, high-amplitude spikes become more prominent, indicating intermittent high-energy collisions with the drum wall.

These observations are quantitatively supported by the summary metrics in Figure 16b. The root mean square dynamic acceleration increases from 0.585 g at 16 RPM to 0.609 g at 18 RPM and 0.633 g at 20 RPM, averaged over the two runs at each speed, indicating a gradual rise in motion intensity.

The peak acceleration values remain consistently high across all speeds, confirming the presence of transient impact events. The relatively small variation in mean acceleration suggests that the overall motion energy is governed by intermittent impacts rather than sustained high acceleration levels.

The gyroscope range of ±2000°/s is reached by 3.9 to 5.6% of samples in each run, so angular rate statistics are censored at the upper end, and the reported peak and 95th-percentile angular velocities are lower bounds. A wider-range gyroscope would be required to characterise the extremes of this motion.

The angular velocity signals shown in Figure 16c further illustrate the rotational dynamics of the particle. The measured angular velocity fluctuates around zero due to continuous reorientation of the particle during motion. At lower speeds, the signal remains relatively compact, while higher speeds result in increased spread and more frequent extreme values. This behaviour reflects a transition from predominantly rolling motion to more irregular and energetic movement regimes within the drum.

Overall, the time domain analysis demonstrates that increasing rotational speed primarily influences the variability and intermittency of particle motion rather than significantly altering the average energy level. The observed behaviour reflects a transition toward more dynamic and impact-dominated motion at higher speeds, which can influence both mechanical response and wireless signal stability.

#### 4.3.2. Frequency Characteristics of Particle Motion

**Figure 17:**
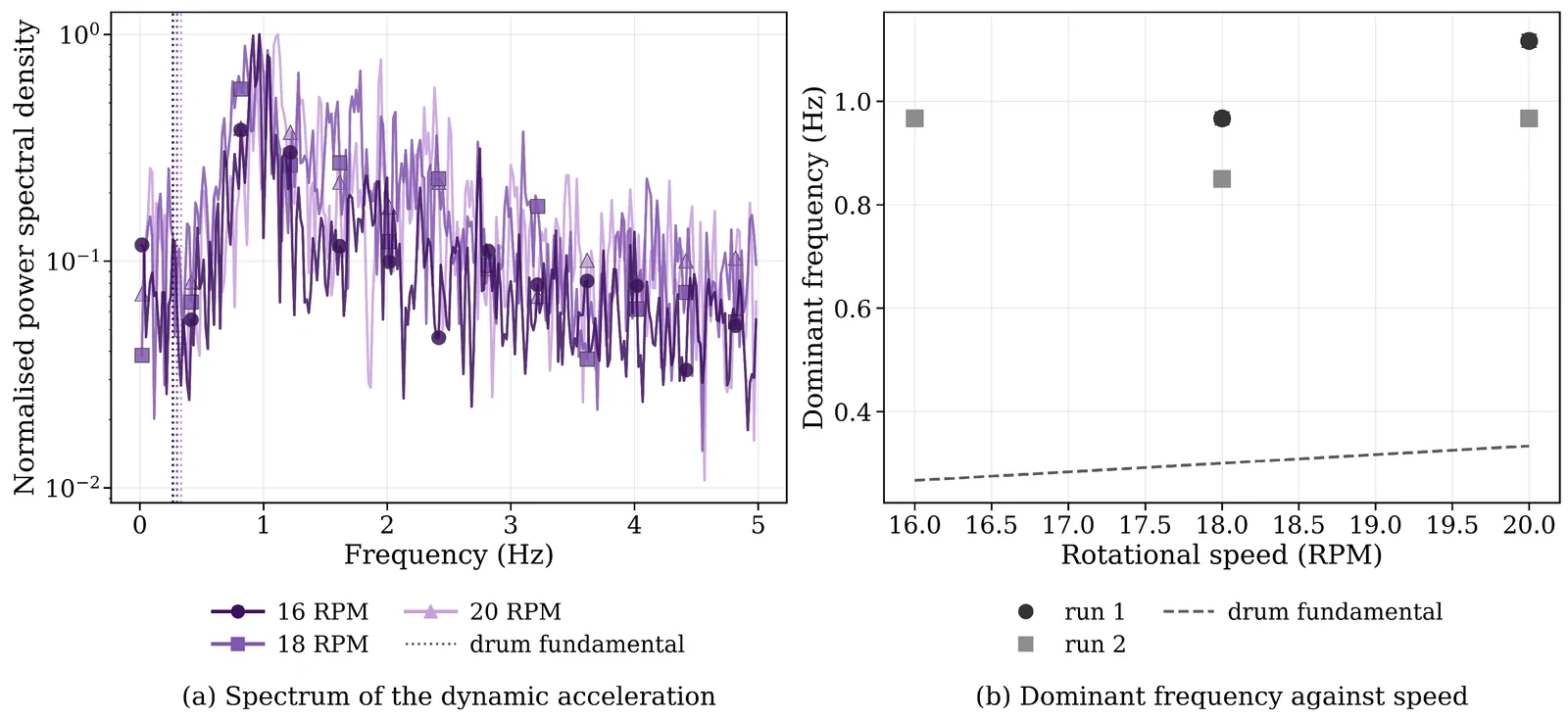
(**a**) Power spectral density of the dynamic acceleration with the estimation parameters annotated. (**b**) Dominant frequency per run against rotational speed, with error bars of half the resolution bandwidth.

The frequency characteristics of the smart particle motion were analysed using spectral analysis of the gyroscope signals. The resulting spectra, as illustrated in Figure 17, show that most of the motion energy is concentrated in the low-frequency region, which reflects the periodic behaviour imposed by the rotating drum. This consistent dominance of low-frequency components across all operating conditions indicates that the particle motion is primarily governed by the external rotational forcing rather than by rapid internal variations (Figure 17a).

Spectra are estimated by Welch’s method, with a Hann window, 6000-sample segments (60 s) at 50% overlap, linear detrending, and no zero padding, giving a bin spacing of 0.0167 Hz and a resolution bandwidth of 0.025 Hz. The dominant frequency is identified as the highest-power peak within 0.05–5.0 Hz whose prominence exceeds 10% of the in-band maximum. On this criterion, the per-speed mean dominant frequencies are 0.967 Hz at 16 RPM, 0.908 Hz at 18 RPM, and 1.042 Hz at 20 RPM, as shown in Figure 17b. The range across speeds, 0.133 Hz, is smaller than the run-to-run spread at a single speed, 0.150 Hz, and the rank correlation between speed and dominant frequency is ρ = 0.00 (*p* = 1.00). The dominant frequency of particle motion is therefore not resolvable by being dependent on rotational speed over the range tested. It sits at approximately three times the drum fundamental in every run, indicating that the particle is not rigidly carried with the shell but is repeatedly lifted and released, consistent with the lifter passage frequency. However, the overall distribution of spectral energy remains largely unchanged. The proportion of energy contained within the low-frequency range remains similar across operating conditions, and the spectral centroid shows only minor variation. This suggests that while the motion becomes more active, its underlying frequency structure is preserved.

### 4.4. Motion Intensity at Different Rotational Speeds

**Figure 18:**
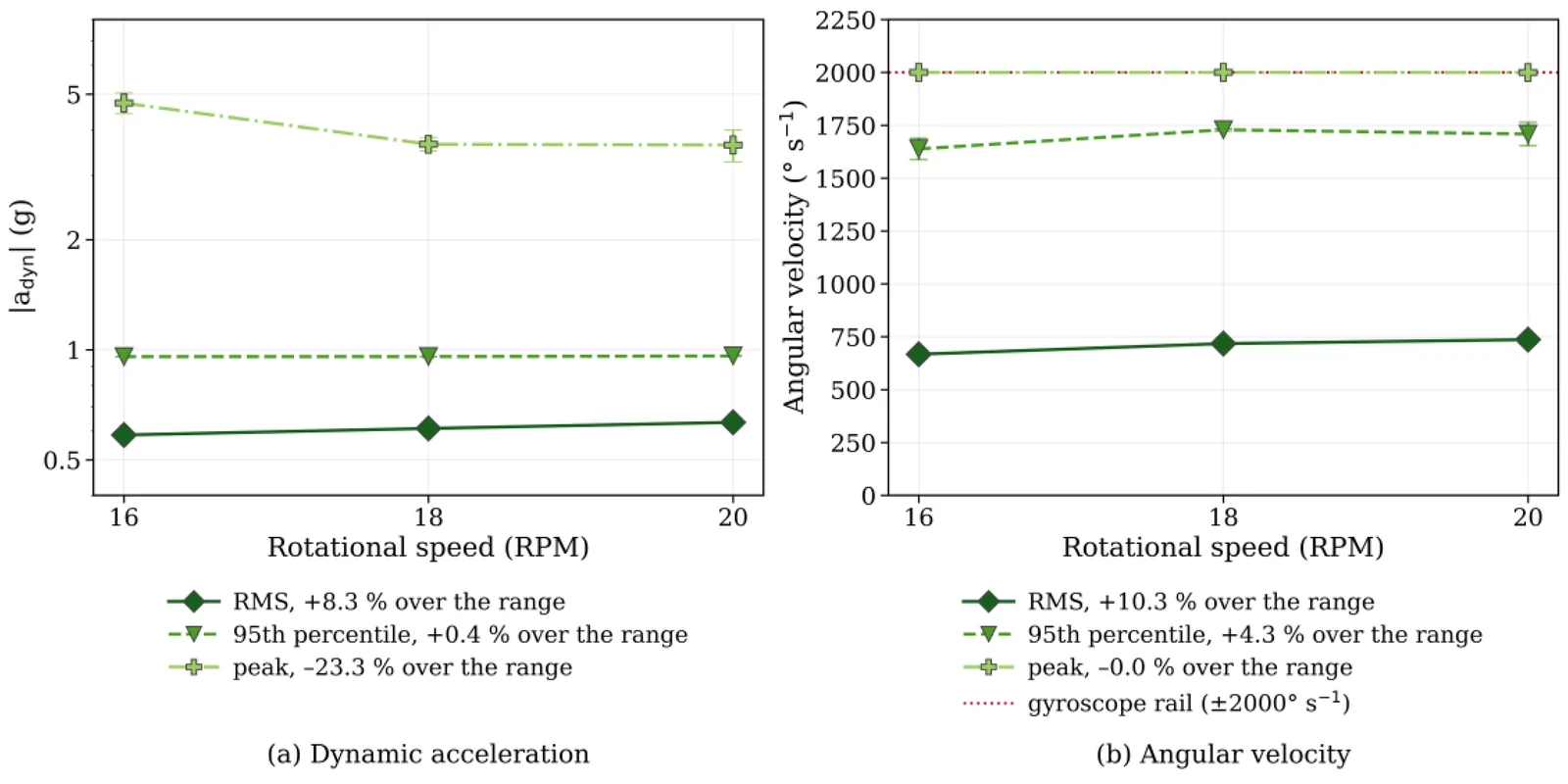
Motion intensity metrics derived from IMU measurements at different rotational speeds, including RMS, P95, and peak values for (**a**) dynamic acceleration and (**b**) angular velocity.

Figure 18 presents the variation of motion intensity metrics across different rotational speeds. A consistent increase in RMS values is observed for both acceleration and angular velocity as the rotational speed increases from 16 RPM to 20 RPM. This trend indicates a progressive increase in the overall energy and activity of particle motion, reflecting stronger inertial forces and larger excursions within the rotating system.

The P95 metric follows a similar increasing trend, capturing the upper range of motion while remaining less sensitive to isolated spikes. This behaviour demonstrates that the increase in motion intensity is not limited to rare events but is representative of the general dynamic behaviour of the particle. As such, P95 provides a robust indicator of high-intensity motion while maintaining stability against noise and outliers.

In contrast, the peak values do not exhibit a strictly monotonic trend. While peak angular velocity and acceleration remain high across all rotational speeds, slight variations are observed between operating conditions. This behaviour reflects the influence of intermittent contact dynamics and localised variations in motion, where extreme values are governed by instantaneous interactions rather than sustained motion patterns.

A comparison between acceleration and angular velocity metrics further highlights the sensitivity of gyroscope measurements to changes in rotational speed. The increase in RMS and P95 angular velocity is more pronounced compared to acceleration, indicating that angular motion provides a clearer representation of rotational dynamics within the system.

Overall, the combined analysis of RMS P95 and peak metrics demonstrates that IMU-derived features can effectively characterise motion intensity under varying operating conditions. The results align with the expected physical behaviour of the system and support the use of embedded IMU sensing for monitoring particle dynamics in rotating machinery. This capability is essential for applications where direct observation is not feasible, enabling data-driven analysis and potential integration into condition monitoring and digital twin frameworks.

### 4.5. Coupling Between Wireless and Motion Signals

#### 4.5.1. Resolution of the Measurement

Wireless and inertial measurements were acquired on independent clocks, so the analysis proceeds at the ensemble level using the drum revolution as the common reference. A second constraint applies irrespective of synchronisation. The receiver logged at a median rate of 0.98 Hz, giving a Nyquist frequency of 0.49 Hz. The drum fundamental spans 0.267 to 0.333 Hz and is therefore resolved at 2.9 to 3.7 samples per revolution. The dominant motion component, at 0.91 to 1.04 Hz, lies above the Nyquist limit and is aliased in the wireless record. Sample-level coupling between received power and motion is consequently not recoverable at this logging rate by any post-processing. Joint wireless–motion measurement in rotating machinery requires the received-power stream to be logged at a rate resolving the motion band, here at least 5 Hz, on a clock shared with the inertial unit.

#### 4.5.2. Rotation-Locked Modulation

Both streams are folded onto a rotation phase, φ = (t f_0_) mod 1, with f_0_ = N/60 for a drum speed N in rev/min. Modulation at the rotation frequency is tested by fitting

(9) $$y\left(t\right)=a_{0}+\sum_{h=1}^{2}\left[a_{h}\cos\left(2\pi hf_{0}t\right)+b_{h}\sin\left(2\pi hf_{0}t\right)\right]+\varepsilon \left(t\right)$$

to each raw, irregularly sampled series by least squares, with an F-test against the null of no modulation. Fitting the raw series avoids interpolating a record sampled at about 1 Hz, which would introduce structures not present in the data. The modulation amplitude at the fundamental is (a_1_^2^ + b_1_^2^)^½^.

The motion-intensity envelope is significantly modulated at f_0_ in all six drum runs, confirming that the rotation is imprinted on the motion. In the wireless records, a significant rotation-locked component is detected in one of nine sensor–speed combinations, with a maximum modulation amplitude of 0.92 dB. Coupling between rotation phase and received power is therefore weak and not consistent across conditions. This is not a range effect: as established in Section 3.1, the receiver lies on the axis of rotation, so the geometric path length to a particle at fixed radius is invariant under rotation. Any modulation must arise from antenna orientation, shadowing by the lifters and shell, or multipath.

**Figure 19:**
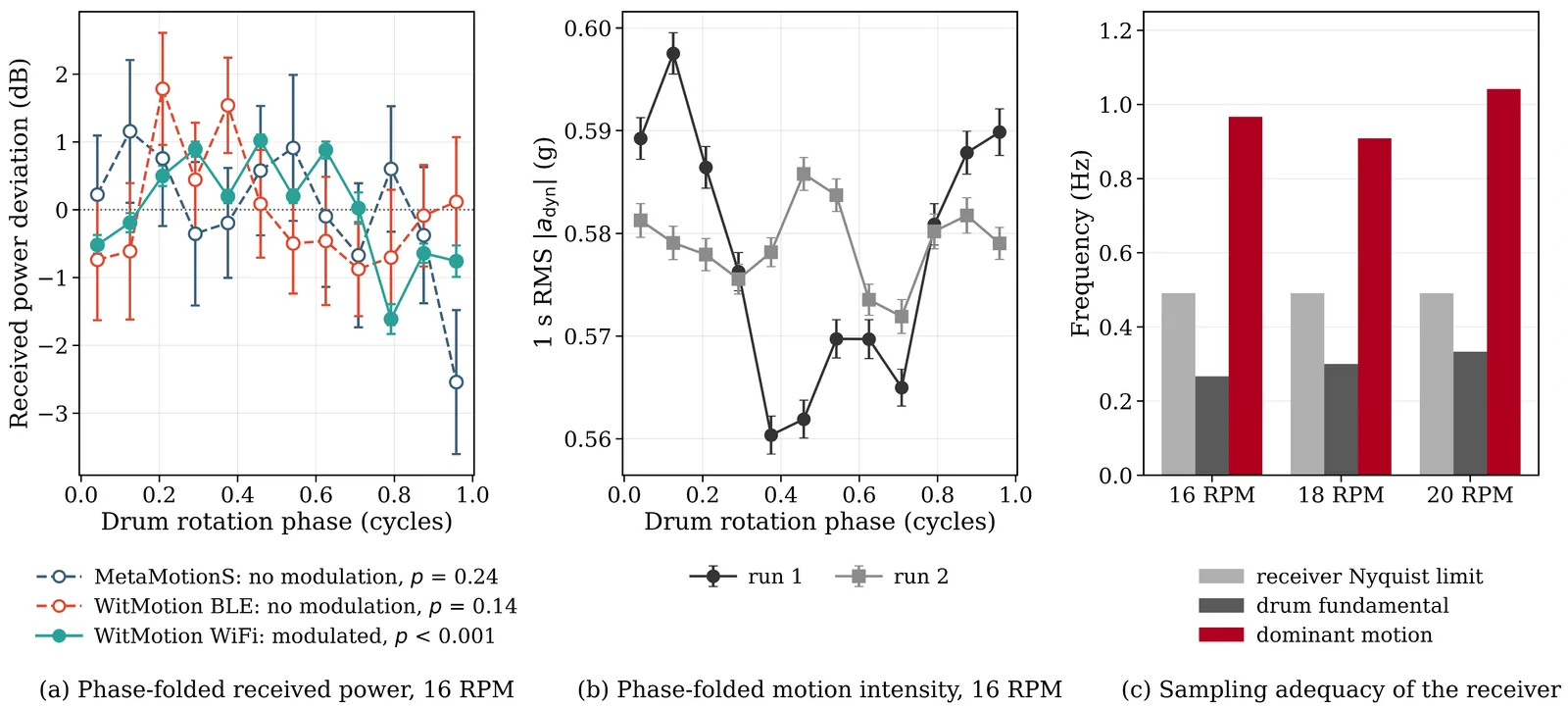
Ensemble coupling between the wireless and motion streams. (**a**) Received power folded onto the drum rotation phase at 16 RPM, with the standard error of each phase bin; solid lines with filled markers indicate significant modulation at the rotation frequency, dashed lines with open markers indicate no significant modulation. (**b**) The same fold applied to the motion–intensity envelope for both runs at that speed. (**c**) Receiver Nyquist limit against the drum fundamental and the observed dominant motion frequency; the motion band lies above the Nyquist limit at every speed.

Because the two campaigns have independent phase origins, the relative phase between the wireless and motion profiles is not identifiable, and only the presence, amplitude, and significance of the modulation are interpreted, as shown in Figure 19.

#### 4.5.3. Orientation and Motion Regime

**Figure 20:**
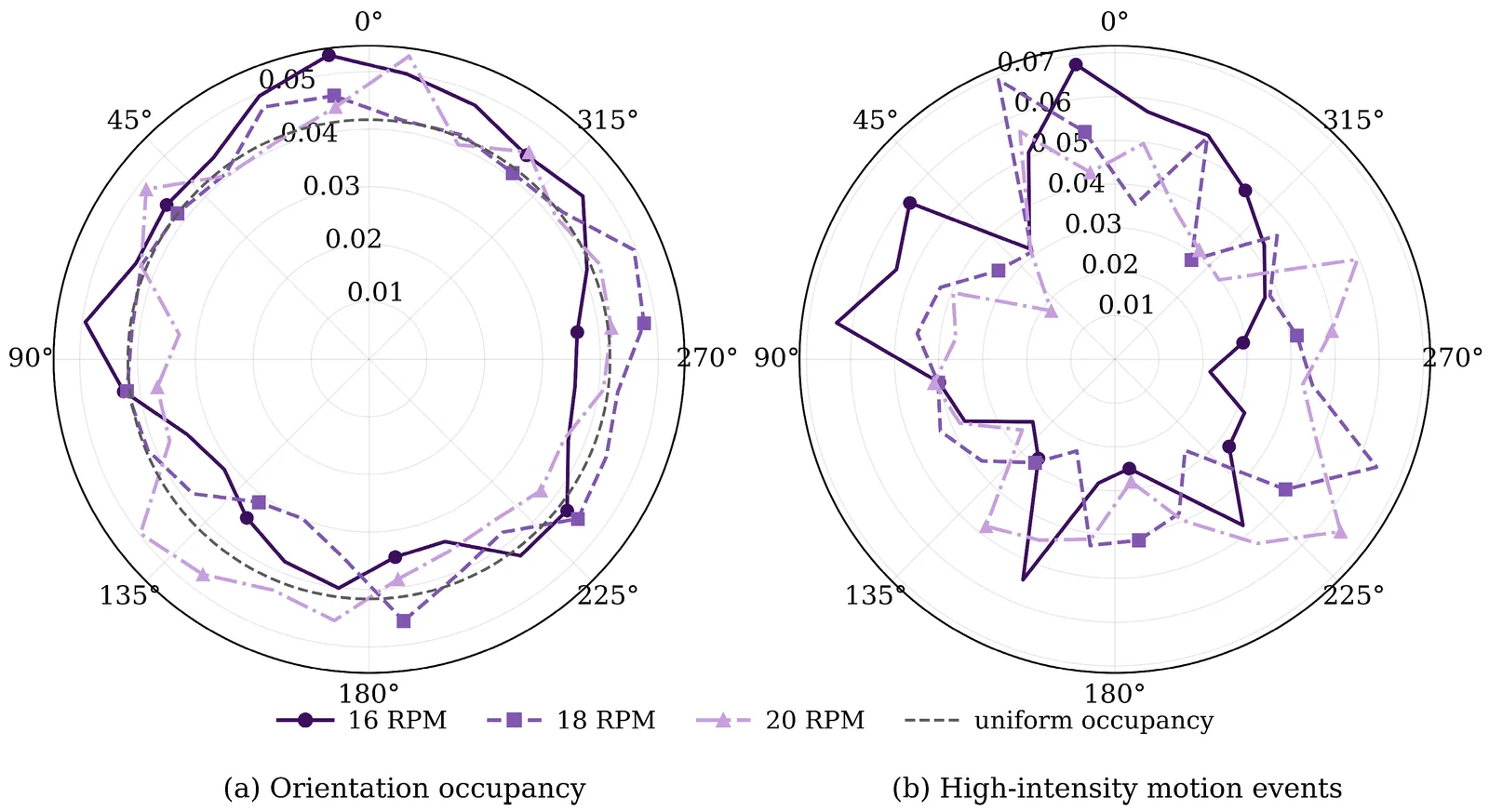
Orientation structure of the particle motion. (**a**) Polar occupancy of roll angle about the drum axis, with the uniform reference shown as dashed lines. (**b**) Polar distribution of high-intensity motion events.

Orientation occupancy about the drum axis is statistically indistinguishable from uniform at all three speeds, while high-intensity motion events, defined as the upper 5% of the 1 s root mean square dynamic acceleration envelope, are significantly concentrated relative to occupancy, as shown in Figure 20. The test statistics are: 16 RPM, Rayleigh *p* = 0.44 for occupancy and χ^2^ = 59.0 (*p* = 5.4 × 10^−5^) for events against occupancy; 18 RPM, Rayleigh *p* = 0.91 for occupancy and χ^2^ = 37.5 (*p* = 2.9 × 10^−2^) for events against occupancy; and 20 RPM, Rayleigh *p* = 1.00 for occupancy and χ^2^ = 54.8 (*p* = 2.1 × 10^−4^) for events against occupancy. Impact-like events therefore occur preferentially at orientations even though the particle distributes its time uniformly over orientation, which identifies a testable mechanism for orientation-dependent fading. Characterising that mechanism requires received power and orientation to be recorded on a common clock and is identified as the principal direction for further work.

#### 4.5.4. Non-Stationarity of Signal Variability

A single standard deviation over an entire record conceals variation in the motion regime. A sliding 31-sample standard deviation varies by a factor of 1.7 to 6.2 within individual records, and a Brown–Forsythe test comparing the first, middle, and final thirds rejects constant variance in three of twelve records. With a single particle in a bare drum, the classical charge motion phases are not well defined, and the wireless samples cannot be labelled by motion phase because the streams are not simultaneous; the inertial records are instead segmented into low-, medium-, and high-intensity regimes by terciles of the envelope.

## 5. Discussion

### 5.1. Sensor Selection and Communication Performance

The performance of the smart particle is influenced not only by motion sensing capabilities but also by the reliability of wireless data transmission. The RSSI analysis shows that signal strength varies with operating conditions, with increased fluctuations observed under rotational motion. These variations are primarily caused by changes in sensor orientation and partial obstruction within the rotating environment.

Among the evaluated sensors, Wi-Fi-based communication demonstrates more stable RSSI behaviour compared to BLE, particularly at higher rotational speeds. This improved stability enables more reliable data transmission during dynamic motion and supports the continuous monitoring of particle behaviour. These observations highlight the importance of sensor selection in ensuring both measurement accuracy and communication robustness in smart particle systems.

The stability advantage is a property of the device, antenna, and enclosure combination rather than of the communication protocol. The two BLE particles differ from one another by 23.9 dB in mean received power under rotation, a separation far larger than any protocol-class effect, and two radios exposed to the same channel realisation at the same instant experience uncorrelated fading. Performance differences are accordingly attributed to the combination of device, antenna, and protocol rather than to the protocol alone.

### 5.2. Particle Motion Behaviour

Within the investigated speed range, the particle is carried up the shell by the lifters, released, and falls back onto the wall. At Froude numbers of 0.0429 to 0.0671, well below unity, centrifuging does not occur, and the motion is dominated by gravitational fall and impact. As rotational speed increases, the particle exhibits greater radial displacement and increased variability, indicating more energetic motion. This behaviour is consistent with the IMU-derived motion intensity metrics, where RMS and P95 values increase with rotational speed. These trends confirm that higher operating speeds lead to stronger inertial effects and enhanced particle activity. The results demonstrate that the instrumented particle can reliably capture the motion intensity in rotating systems.

### 5.3. Motion Intensity and Sensor Response

The motion intensity analysis shows that RMS and P95 values increase consistently with rotational speed, indicating a clear rise in sustained particle activity. In contrast, peak values show non-monotonic behaviour due to localised transient effects. This suggests that RMS and P95 provide more reliable indicators of overall motion behaviour than peak-based metrics. In addition, angular velocity measurements show greater sensitivity to changes in operating conditions than acceleration, indicating that gyroscope data provides a more direct representation of rotational dynamics.

### 5.4. Limitations and Future Work

The present study demonstrates the feasibility of using an instrumented particle for motion sensing and validation in a controlled rotating system. The experiments conducted under well-defined conditions enable reliable evaluation of sensing performance and provide clear insight into particle motion behaviour. These results establish a foundation for extending smart particle sensing approaches to more complex rotating equipment, such as tumbling mills and granulation drums, where particle motion plays a key role in process efficiency. The limitations are as follows:

- The drum was operated bare, with a single unconstrained particle. Trajectories are not repeatable, and the results characterise a baseline rather than a charged mill;
- Three speeds were tested, spanning 21 to 26% of critical speed. The regime remains well below centrifuging, and the findings should not be extrapolated to higher speeds;
- Wireless and inertial measurements were acquired on independent clocks, which restricts the coupling analysis to the ensemble level;
- At approximately 1 Hz, the receiver resolves the drum fundamental but not the dominant motion band, which is aliased. This is a limit of the measurement design rather than of the analysis;
- Between 3.9 and 5.6% of drum samples reach the ±2000°/s gyroscope range, so extreme angular rates are censored;
- The optical reference used for inertial validation cannot follow the bob above about 758°/s, so validation is established in the small-amplitude regime (median local |r| = 0.96) and not across the full swing;
- No radiation pattern characterisation was performed. A bench turntable measurement of each particle in its enclosure is identified as the appropriate means of establishing the orientation dependence suggested by the results of Section 4.5.3;
- Absolute received power is limited by the receiver’s quantisation and by the absence of a reference transmitter calibration. Any residual offset affects absolute values but cancels out in the relative comparisons and variance-based metrics on which the conclusions rest.

While the current framework focuses on a single particle, it provides a simplified representation of the system that is well suited for the initial validation of sensing and reconstruction methods. In practical systems, particle motion is influenced by interaction effects, collective behaviour, and energy transfer within a granular bed. Future studies will therefore incorporate granular media to investigate these interaction-driven dynamics and better represent realistic operating conditions.

Further work will also focus on improving trajectory mapping accuracy through the integration of synchronised vision tracking with IMU measurements. This will enable the development of advanced sensor fusion approaches for more accurate motion estimation. In addition, multi-particle sensing and a wider range of operating conditions will be explored to capture diverse flow regimes and enhance the applicability of smart particle systems for industrial monitoring and digital twin applications in rotating machinery.

A synchronised re-acquisition, in which received power and inertial data are logged on a common clock at a rate resolving the motion band, together with at least one partially charged condition, would convert the ensemble coupling result of Section 4.5 into a sample-level one and address the bare drum limitation in a single campaign.

## 6. Conclusions

This study investigated particle dynamics in a rotating drum using a smart particle equipped with inertial sensing capabilities. The proposed approach enabled the analysis of motion intensity using IMU-derived features under different rotational speeds. In addition, the performance of wireless communication was evaluated through RSSI analysis to assess sensor reliability in dynamic environments.

The results show that the motion of an unconstrained single particle in a lifter-equipped bare drum becomes more variable and more impact-dominated as rotational speed increases, without a resolvable shift in its dominant frequency. The analysis of motion intensity metrics indicates that RMS and P95 values increase consistently with rotational speed, providing reliable indicators of sustained motion behaviour, while peak values exhibit non-monotonic variation due to transient effects. Once transmit power is accounted for, the wireless results are best expressed as a device-level rather than a protocol-level finding: two BLE particles differ from one another by 23.9 dB, and simultaneously logged particles exhibit uncorrelated fading, so the stability ranking reflects the particle implementation rather than the choice between Wi-Fi and BLE. Choosing between them further depends on the weight given to endurance, with the Wi-Fi particle costing 8.0 times as much energy per delivered packet as the most efficient candidate.

Importantly, the results establish a practical basis for sensor selection, where communication stability and signal consistency are considered alongside motion-sensing capability for the reliable deployment of smart particles in rotating environments. Overall, the findings demonstrate that instrumented smart particles provide an effective tool for investigating particle motion inside rotating process equipment. This approach offers strong potential for monitoring and optimizing industrial systems such as granulators, mixers, and tumbling mills.

Finally, this study establishes a measurement design requirement for work of this kind. Coupling between wireless and motion signals in a rotating machine is recoverable at the sample level only if the received power stream is logged on a clock shared with the inertial unit and at a rate that resolves the motion band, which for the drum studied here means at least 5 Hz. At the cadence used, only ensemble-level, rotation-phase-resolved coupling is recoverable, and it is weak: a rotation-locked component was detected in one of nine sensor–speed combinations. Reporting this bound defines the instrumentation required for the integrated analyses that this field has so far assumed to be available.

## Figures and Tables

**Table 1:** Comparison of existing studies on rotating machine monitoring and the proposed smart-particle sensing framework.

Refs.	Monitoring Approach	Application Environment	Wireless Signal Analysis	Motion Measurement	Motion Signal Coupling	Integrated Wireless and Motion Analysis
[10,11,12]	Analytical and physics-based models	Rotating electrical machines	No	Indirect	No	No
[13]	Vibration-based monitoring	Industrial rotating machines	No	Indirect	No	No
[14]	Wireless instrumented sphere	Rotating bodies	Not primary	Acceleration sensing	No	No
[15,16]	Instrumented grinding media	Tumbling mills and rotating process equipment	No	Acceleration sensing	No	No
[17]	Wireless propagation analysis	Metallic and enclosed environments	Yes	No	No	No
[18]	Wireless in metallic environments	Metal enclosures	Yes	No	No	No
[19,20]	RSSI-based wireless link analysis	Wireless sensor networks	Yes	No	No	No
[21]	Wireless sensing in rotating machines	Rotating electrical machines	Yes	No	Partial	No
[22,23]	IMU-based motion sensing	General dynamic systems	No	Yes	No	No
[24,25,26,27,28,29,30]	Granular motion analysis (experimental or simulation)	Rotating drums and granular systems	No	Yes	No	No
This work	Smart particle sensing with wireless RSSI analysis and IMU motion measurement	Rotating drum	Yes	Yes	Partial (rotation phase resolved)	Partial (rotation phase resolved)

**Table 2:** Testbed specification and operating regime. Froude numbers are given both for the bare shell radius and for the lifter crest radius on which a carried particle sits.

Parameter	Value
Internal diameter	300 mm
Internal depth	310 mm
Internal volume	21.9 L
Lifters	6 × triangular, 7 mm high, 60° apart
Lifter crest radius	143 mm
Critical speed	77.2 RPM
Speeds tested	16, 18, 20 RPM
Percentage of critical speed	20.7%, 23.3%, 25.9%
Froude number (shell)	0.0429, 0.0543, 0.0671
Froude number (lifter crest)	0.0409, 0.0518, 0.0639
Charge	none; single instrumented particle
Receiver position	on the axis of rotation, 80 mm beyond the end plate
Particle-to-receiver distance	164–415 mm (275 mm at mid-depth)
Free-space path loss at mid-depth	29.0 dB at 2.44 GHz
Drum shell material and wall thickness	Mild Steel
End-plate material	Perspex

**Table 3:** Technical specifications of the smart particle sensors evaluated.

Specification	MetaMotionS	Witmotion BLE	Witmotion WIFI
Communication	BLE 2.4 GHz	BLE 5.0, GATT over L2CAP	WIFI 2.4 GHz (TCP/UDP)
Accelerometer Range	±2 g, ±4 g, ±8 g, ±16 g	±16 g	±16 g
Accel Resolution	2048 to 16,384 counts/g	2000 counts/g (0.0005 g/LSB)	2000 counts/g (0.0005 g/LSB)
Gyroscope Range	±125 to ±2000°/s	±2000°/s	±2000°/s
Gyro Resolution	16 to 262 counts/°/s	16.39 counts/°/s (0.061°/s/LSB)	16.39 counts/°/s (0.061°/s/LSB)
Magnetometer Range	±12 to ±13 Gauss (±1200 to ±1300 µT)	±2 Gauss	±30 Gauss
Max Data Rate	Accel: 1600 Hz, Gyro: 3200 Hz	Output Rate: 100 Hz	Output Rate: 100 Hz
Additional Sensors	Barometer, Ambient Light, Thermistor	None explicitly	Temperature
Operating Temp	−40 °C to 85 °C	−20 °C to 60 °C	−20 °C to 70 °C
Local Storage	4 Gbit SLC NAND Flash	None	None
**PHY/link standard**	BLE, 2.4 GHz	BLE, 2.4 GHz	IEEE 802.11 b/g/n [35], 2.4 GHz
**Transport/profile**	GATT over L2CAP	GATT over L2CAP	TCP/UDP
**Radio system-on-chip**	Nordic nRF52840	Nordic nRF52832	Espressif ESP32
**Transmit power**	0 dBm (firmware default)	≤4 dBm	≤20.0 dBm
**Antenna type**	integrated PCB antenna	integrated module antenna	integrated PCB antenna
**Battery capacity**	100 mAh	250 mAh	240 mAh

Note: MetaMotionS sensors were sourced from MbientLab Inc. (San Francisco, CA, USA). WitMotion BLE and Wi-Fi sensors were sourced from WitMotion Shenzhen Co., Ltd. (Shenzhen, China). Nordic nRF52840 and nRF52832 system-on-chips are manufactured by Nordic Semiconductor (Trondheim, Norway). Espressif ESP32 system-on-chips are manufactured by Espressif Systems (Shanghai, China).

**Table 4:** Packet delivery statistics are reconstructed from the receiver logs.

Sensor	Condition	Cadence (ms)	Expected	Delivered	Loss (%)	Loss 95% CI	Dropouts	IPI p95 (ms)	Excluded Tail
MetaMotionS	Stationary	1018	538	510	5.20	[3.62, 7.42]	22	1020	0
WitMotion BLE	Stationary	1023	591	591	0.00	[0.00, 0.65]	0	1026	0
WitMotion WiFi	Stationary	2991	500	500	0.00	[0.00, 0.76]	0	2991	0
MetaMotionS	16 RPM	1018	529	528	0.19	[0.03, 1.06]	1	1019	0
WitMotion BLE	16 RPM	1022	607	607	0.00	[0.00, 0.63]	0	1025	0
WitMotion WiFi	16 RPM	1022	514	514	0.00	[0.00, 0.74]	0	1025	93
MetaMotionS	18 RPM	1018	513	508	0.97	[0.42, 2.26]	5	1020	0
WitMotion BLE	18 RPM	1020	511	511	0.00	[0.00, 0.75]	0	1023	0
WitMotion WiFi	18 RPM	1020	500	500	0.00	[0.00, 0.76]	0	1023	11
MetaMotionS	20 RPM	1019	521	520	0.19	[0.03, 1.08]	1	1020	0
WitMotion BLE	20 RPM	1020	520	520	0.00	[0.00, 0.73]	0	1023	0
WitMotion WiFi	20 RPM	1020	500	500	0.00	[0.00, 0.76]	0	1023	20

**Table 5:** Received-power statistics by particle and condition, with effective sample size, confidence intervals on both the mean and the standard deviation, and link margin referenced to the receiver sensitivity.

Sensor	RPM	n	n_eff_	Mean RSSI (dBm)	95% CI	SD (dB)	SD 95% CI	Median	IQR (dB)	Link Margin (dB)
MetaMotionS	0	510	97	−87.86	[−88.10, −87.62]	1.19	[0.98, 1.46]	−88.0	1.8	6.1
WitMotion BLE	0	591	64	−53.31	[−53.69, −52.93]	1.52	[1.40, 1.63]	−53.0	2.0	40.7
WitMotion WiFi	0	500	16	−43.95	[−44.55, −43.35]	1.14	[1.00, 1.22]	−44.0	2.0	53.0
MetaMotionS	16	528	494	−82.59	[−83.17, −82.00]	6.60	[6.30, 6.90]	−83.0	10.0	11.4
WitMotion BLE	16	607	411	−58.54	[−59.13, −57.96]	6.04	[5.73, 6.37]	−58.0	10.0	35.5
WitMotion WiFi	16	514	261	−47.23	[−47.39, −47.08]	1.27	[1.00, 1.58]	−47.0	2.0	49.8
MetaMotionS	18	508	448	−82.37	[−82.97, −81.77]	6.46	[6.14, 6.77]	−82.0	10.0	11.6
WitMotion BLE	18	511	233	−57.96	[−58.80, −57.12]	6.52	[5.93, 7.20]	−56.0	9.0	36.0
WitMotion WiFi	18	500	110	−46.98	[−47.37, −46.60]	2.03	[1.73, 2.37]	−47.0	2.0	50.0
MetaMotionS	20	520	448	−81.55	[−82.17, −80.93]	6.68	[6.35, 6.99]	−81.0	10.0	12.4
WitMotion BLE	20	520	387	−58.37	[−58.99, −57.74]	6.25	[5.90, 6.60]	−57.0	10.0	35.6
WitMotion WiFi	20	500	38	−46.90	[−47.75, −46.05]	2.59	[2.33, 2.84]	−47.0	3.0	50.1

**Table 6:** Power, endurance, and energy per successfully delivered packet. Capacities and operating currents are manufacturers’ published figures; delivered packet rates are measured in this study.

Sensor	Radio SoC	Battery (mAh)	Mean Current (mA)	Run Time (h)	Delivered Rate (Hz)	Packets Per Charge	Energy Per Packet (mJ)	Relative Cost
MetaMotionS	Nordic nRF52840	100	10	10.0	0.978	35,192	37.9	1.00×
WitMotion BLE	Nordic nRF52832	250	25	10.0	0.980	35,271	94.4	2.49×
WitMotion WiFi	Espressif ESP32	240	80	3.0	0.980	10,581	302.1	7.98×

**Table 7:** Motion statistics by speed and run, reported per run so that run-to-run variability remains visible.

RPM	Run	fs (Hz)	Duration (s)	Gyro Saturated (%)	RMS |a_dyn_| (g)	P95 |a_dyn_| (g)	RMS |ω| (°/s)
16	1	100	263	4.03	0.585	0.958	1027
16	2	100	336	4.26	0.585	0.958	1008
18	1	100	292	5.22	0.610	0.958	1131
18	2	100	311	3.89	0.609	0.959	1080
20	1	100	312	5.57	0.635	0.960	1220
20	2	100	271	4.89	0.631	0.964	1172

## Data Availability

The data presented in this study are available on request from the corresponding author.

## Abbreviations

The following abbreviations are used in this manuscript:

RSSI	Received Signal Strength Indicator
IMU	Inertial Measurement Unit
RMS	Root Mean Square
P95	95th Percentile
FFT	Fast Fourier Transform
BLE	Bluetooth Low Energy
EIRP	Equivalent Isotropically Radiated Power
FSPL	Free-Space Path Loss
CI	Confidence Interval
SD	Standard Deviation
IQR	Interquartile Range
TOPSIS	Technique for Order of Preference by Similarity to Ideal Solution
PSD	Power Spectral Density
QoS	Quality of Service
IPI	Inter-Packet Interval
Fr	Froude number
